# Oral parasites alter male advertisement signals and female mate choice in the Green Treefrog (*Dryophytes cinereus*)

**DOI:** 10.1093/cz/zoag003

**Published:** 2026-01-22

**Authors:** Sarah R Goodnight, Ellen F Titus, Michael W McCoy

**Affiliations:** Department of Biology, East Carolina University, 101 E 10th St Suite 2500, Greenville, NC 27858, USA; The Nature Conservancy, Minnesota-Dakotas Chapter, 1101 West River Parkway Suite 200, Minneapolis, MN 55415, USA; Department of Biology, Florida Atlantic University: Harbor Branch Oceanographic Institute, 5600 US 1, Fort Pierce, FL 34946, USA

**Keywords:** acoustic signals, digenean, green treefrog, mate choice, parasite, trophic transmission

## Abstract

Parasite infection can impact sexual signals such as advertisement calls through multiple mechanisms and is usually expected to negatively correlate with signal quality. However, many parasites infect their hosts by exploiting predator–prey relationships, thus some aspects of mate quality (i.e., body size, foraging ability) positively covary with infection risk. Here, we employ a system of oral frog tongueworm parasites (*Halipegus occidualis*) and Green Treefrog hosts (*Dryophytes cinereus*) to test the hypothesis that trophically transmitted parasites influence acoustic signals and mate choice in a natural population. Calling male frogs were recorded, parasites in the mouth were counted, and data on call characteristics were extracted using Audacity software and the R package warbleR. Males with high infection abundances produced calls that were shorter and had lower frequencies and different pulse structures than calls produced by uninfected or mildly infected males. Two-choice playback experiments revealed that females selected uninfected male calls only when highly infected male calls were the alternative; however, calls from males infected with a moderate number of worms were selected over those from uninfected males, indicating that the relationship between male signal quality and trophically transmitted parasite infection abundance is nonlinear and complex. Parasite infection generally altered expected relationships between some male traits (call frequency in kHz, call rate, body size) and mate choice, indicating parasite avoidance by females may alter how sexual selection acts on particular call traits and lead to complex patterns in mate choice.

## Graphical Abstract

**Graphical Abstract:**
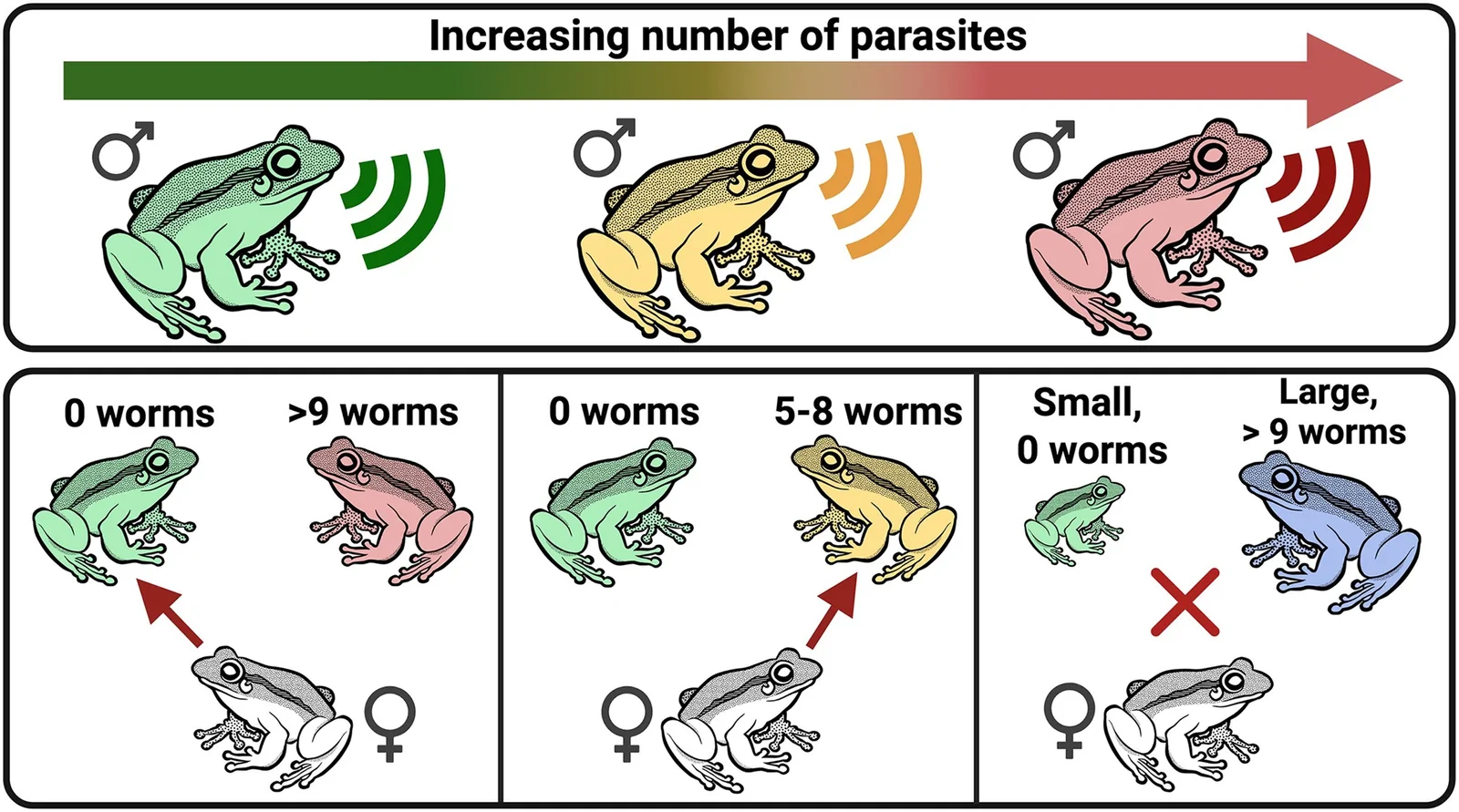

Many species use acoustic signaling for species recognition, territory defense, and mate attraction (Jones 1997; Bee 2015; Mikula et al. 2021). Acoustic advertisement signals play an outsized role in mate choice across a myriad of taxa, including birds (Searcy and Andersson 1986; Byers and Kroodsma 2009; Mikula et al. 2021), primates (Hauser 1993; Fitch and Hauser 1995; Fischer et al. 2004; Delgado 2006), insects (Bentsen et al. 2006; Blankers et al. 2017), fish (Fine and Lenhardt 1983; Amorim et al. 2015; Van Wert and Mensinger 2019), and amphibians (Searcy and Andersson 1986; Gerhardt 1994a; Bee 2015). Complex vocal signals consist of multiple acoustic components that relay various information to potential competitors and mates. For example, the advertisement calls of male túngara frogs (*Engystomops pustulosus*) have 2 parts, a whine and a chuck (Drewry et al. 1982; Gridi-Papp et al. 2006; Ryan and Guerra 2014). Females prefer more complex calls that have a whine and at least one chuck, but male *E. pustulosus* produces whines only in conditions of less competition, increasing the number of chucks with increasing competition for mates (Rand and Ryan 1981; Ryan and Rand 2003; Gridi-Papp et al. 2006; Ryan and Guerra 2014). Not only is the presence of chucks in the call important for female choice but also their frequency; females prefer calls with lower-frequency chucks, which are generally produced by larger males (Ryan 1980, 1983, 1990).

Reliance on complex acoustic signals for broadcasting information about individual status to competitors and mates often leads to selective pressure on specific aspects of calls that are correlated with potential fitness (Ryan and Keddy-Hector 1992; Jones 1997; Fischer et al. 2004). For example, across multiple taxa, dominant frequency covaries with body size (Fine and Lenhardt 1983; Höbel and Gerhardt 2003; Gingras et al 2013; Humfeld and Grunert 2015; Laird et al. 2016) and is often an indicator of mate status and condition (Fischer et al. 2004; Laird et al. 2016). Similarly, in some bird species, males with larger repertoire sizes (the number of unique syllables and song types produced) are able to defend larger territories, attract more mates, and maintain larger harems (Searcy and Andersson 1986; Byers and Kroodsma 2009). Across the animal kingdom, condition-dependent acoustic signals convey information about the physical size or physiological status of the signaler and thus are key foci for sexual selection.

Parasitism has received considerable attention as a key driver of mate choice behavior, since parasitic infections and associated physiological costs are often revealed by variation in secondary sexual traits, body size, stamina, and intrasexual competitive ability of hosts (Weatherhead 1990; Andersson 1994; Moller et al. 1999; Pfennig and Tinsley 2002). The Hamilton–Zuk parasite sexual selection hypothesis states that negative effects of parasites on the health and fitness of hosts should constrain the expression of secondary sexual traits like acoustic signals, and thus honest signals of condition may influence mate choice decisions (Hamilton and Zuk 1982; Read 1987; Zuk 1992; Molnár et al. 2013). Parasite infection can negatively affect signals via reduced body condition (Sanchez et al. 2018) or immune system activation (Garamszegi 2005; Nouri and Blumstein 2019) of the signaler, and they can also disrupt acoustic communication by physically interfering with signal production or detection. For instance, parasitism of nestling Darwin's finches by fly larvae affects their naris size as adults, which in turn affects adult song quality (Kleindorfer et al. 2019), and male frogs with heavy infections of earworms (*Halipegus eccentricus*) suffer loss of hearing and display reduced phonotactic responses to conspecific calls and other acoustic stimuli (Bevier and Gorman Gelder 2018).

While numerous studies have provided empirical support for the basic tenets of parasite-mediated mate choice across taxa (Hamilton and Zuk 1982; Read 1987; Clayton 1990; Worden et al. 2000; Madelaire et al. 2013; Balenger and Zuk 2014), others have not (Edler et al. 2004; Buczek et al. 2016; Greenspan et al. 2016; Megía-Palma et al. 2021), indicating that the relationship between infection and mate choice is complex and likely parasite- and host-species dependent. One reason for the lack of consensus on the effect of parasites on mate choice may be that parasite life cycle structure and transmission strategy can drive the relationship between mate infection status and signal traits preferred by the choosier sex. For example, much empirical support for parasite avoidance in mate choice is from studies of ectoparasites and pathogens that are vertically or horizontally transmitted (e.g., Hamilton and Zuk 1982; Clayton 1990; Able 1996). In contrast, infection with parasites that are transmitted indirectly by vectors, or by trophic transmission from consuming infected prey, pose no direct cost or risk of infection to the chooser (Able 1996). In addition, there may be no indirect costs to the choosier sex when preferred traits (or the fitness of offspring) are uncorrelated (Hausfater et al. 1990; Henderson et al. 1995; Edler et al. 2004) or even positively correlated (Buczek et al. 2016) with infection status. Although not exclusive to trophically transmitted internal parasites, body condition of an infected signaler may in fact be positively correlated with parasite abundance because mates that are larger (Braicovich et al. 2016; Hayward et al. 2017), more aggressive (Munson et al. 2020), or more efficient foragers (Connors et al. 2010; Comas et al. 2014) are also expected to have higher parasite abundances from consuming infected prey. Consequently, mate choice decisions in vector and trophically transmitted parasite–host systems may not follow expectations of parasite-mediated sexual selection and may even be reversed in some cases, such that the choosy sex shows preference for infected mates because infection covaries with other indicators of condition.

Studies investigating the relationship between parasites and the production or quality of male acoustic signals in amphibians have generated mixed results, with some resolving negative (Madelaire et al. 2013), some positive (An and Waldman 2016), and some equivocal or context-dependent (Hausfater et al. 1990; Greenspan et al. 2016; Moretti et al. 2017; Shangi et al. 2022) relationships. However, most studies focus on directly transmitted pathogens or non-trophically transmitted parasites. Consequently, empirical choice experiments that explicitly test mate choice of advertisement signals from mates infected with trophically transmitted parasites are rare. This study aims to evaluate how infection by a trophically transmitted oral cavity parasite (parasitic tongueworms, *Halipegus occidualis*) affects characteristics of advertisement call production and subsequent mate choice of the Green Treefrog (*Dryophytes cinereus*). Green Treefrogs are a tractable and well-studied model of sexual selection and mate choice for specific aspects of male acoustic advertisement signals (Gerhardt 1987; Höbel and Gerhardt 2003; Christensen-Dalsgaard et al. 2020). Specifically, research on this species suggest that females show a general preference for lower call dominant frequencies, higher call rates, and potentially longer call durations in realistic calls (Gerhardt 1987; Asquith and Altig 1990; Gerhardt 1991; Höbel and Gerhardt 2003; Laird et al. 2016; Leary et al. 2021), which generally correlate with larger male size and/or body condition. Other characters of the Green Treefrog call (e.g., pulse structure) are important for species recognition (Laird et al. 2016). We test whether parasites alter acoustic characteristics of male vocalizations by recording infected and uninfected male Green Treefrog advertisement calls over 3 breeding seasons, and whether parasite infection affects female mate choice by using 2-choice playback experiments in a wild population.

## Study system

In recent decades, the Green Treefrog (*Dryophytes cinereus*, formerly *Hyla cinerea*) has emerged as a general model for the study of signal evolution (Höbel and Gerhardt 2003; Robillard et al. 2006; Buerkle et al. 2014; Bee 2015) and sound recognition physiology (Christensen-Dalsgaard et al. 2020) in amphibians. Male Green Treefrogs form large choruses at breeding ponds and inflate their vocal sacs to produce loud, repeated, “honking” advertisement calls from perches on emergent vegetation. Females show preferences for certain aspects of calls, some of which correlate with male condition and body size (Feng et al. 1976; Perrill et al. 1982; Gerhardt 1987; Gerhardt et al. 1987). Specifically, playback response experiments involving both natural and synthesized calls have identified specific call parameters that drive female preference (Gerhardt 1987, 1991; Neelon et al. 2019). Studies that restrict call parameters to include only realistic values observed in natural populations have shown that females prefer calls with lower dominant frequencies (Asquith and Altig 1990; Gerhardt 1991), longer call durations (Gerhardt 1987; Gerhardt and Huber 2002; Leary et al. 2021), and higher call rates (Gerhardt 1987; Laird et al. 2016) because these traits may signal a higher-quality mate. Call traits like call dominant frequency and duration may be the result of stabilizing rather than directional selection, but when restricted to realistic naturally occurring ranges, lower frequencies and longer duration calls have been shown to be selected by choosy females across several experimental studies over the last 50 years (Gerhardt 1987, 1991; Laird et al. 2016; Leary et al. 2021).

Green Treefrogs serve as definitive hosts for a wide variety of parasites, but we lack a robust understanding of how parasites affect their physiological condition, overall fitness, and reproductive behavior (Creel et al. 2000; Albecker et al. 2018; Edwards and Gordon 2021). *Halipegus* is a genus of digenean trematode parasite with a unique life cycle that includes 4 sequential host species and both sexual and asexual reproductive stages (Thomas 1939; Zelmer and Esch 1998). Frogs obtain these parasites by consuming infected insects (dragonflies and damselflies) (Zelmer and Esch 1998; Bolek et al. 2010; Goodnight and McCoy 2024). The 2 species of *Halipegus* parasites that occur across North America cluster in the buccal cavities (*H. occidualis)* or in the eustachian tubes (*H. eccentricus*) of their definitive frog hosts after undergoing sexual maturation in the gut (Krull 1935; Thomas 1939; Wetzel and Esch 1996; Zelmer and Esch 1998; Bolek et al. 2010). *Halipegus occidualis* (the “frog tongueworm”) tends to attach to the lingual vein underneath the tongue or on the inside of the cheeks of the frog (Krull 1935; Zelmer and Esch 2000; Stigge and Bolek 2016) and are easily visible upon inspection of the frog's open mouth without the use of anesthetic or destructive sampling of frog hosts (Wetzel and Esch 1996; Stigge and Bolek 2016; Goodnight et al. 2025). Tongueworms can be highly prevalent in host populations, and individual frogs can have as many as 40 worms (Zelmer et al. 1999). Therefore, tongueworm parasites and Green Treefrog hosts form an ideal system to investigate how trophically transmitted parasites impact host acoustic signals, mate choice, and selection on male traits.

This study utilizes this host–parasite system to investigate how parasitism by a trophically transmitted parasite affects characteristics of male advertisement calls and subsequent female mate choice. We predict that infection with tongueworms will be positively correlated with male body size, and that high infection abundances will alter some aspects of vocal signals (e.g., call duration, frequency, pulse structure, and rate). We expect female mate choice patterns to reflect a preference for call traits that reflect larger male body size or condition (e.g., lower dominant frequency and higher call rate) as well as uninfected status. To test these hypotheses, we first quantified parasite abundances and call characteristics of calling male frogs in a wild population, and then assessed the effects of infection on female mate choice in the field using an arena with pairwise playback response experiments.

## Materials and methods

### Collection of call recordings

This study took place at a suburban pond in eastern North Carolina known to harbor a high population density of tongueworm parasites. Activities were permitted under ECU IACUC Animal Use Protocol #D357 and North Carolina Wildlife Collection Permit #17-SC00840. Calling male Green Treefrogs were recorded over 3 summers (2019–2021) during peak calling hours after rain events, generally 9 PM–12 AM from May to August each year. Frog calls were recorded using a Sony Linear PCM recorder (model PCM-M10, Sony Corporation) or a Marantz Solid State recorder (model PMD-661 MKIII, Marantz Professional), and handheld Sennheiser shotgun microphones (models ME66 and MK8070).

We used methods similar to Wilczynski et al. 1984 to collect each recording. Specifically, to focus on signals from a specific frog, a microphone was held approximately 15 cm from the snout of an actively calling male frog until 30–60 s worth of calling activity was recorded. We did not observe any changes in calling behavior during recordings. Frogs were then captured by hand, and infection abundance was quantified by inserting a thin spatula into the mouth of each frog to gently pry it open and count the number of adult tongueworms in the buccal cavity. Frogs were weighed, lightly compressed for measurement accuracy and photographed next to a ruler, then released back at the capture site. Photographs were later analyzed using ImageJ (v1.53, Schneider et al. 2012) to quantify snout-vent length (SVL).

Air temperature at the pond was collected using an AcuRite outdoor thermometer at the time of each recording, and barometric pressure data was collected from a nearby weather station (station number CamelotPir8, 35.58 °N, 77.33 °W, distance from site ∼1.4 km) accessed through Weather Underground (TWC Product and Technology LLC 2014). We also recorded the time of night each recording was taken.

### Acoustic analysis

Call recordings were annotated, background noise was removed, and sound quality was evaluated using Audacity software version 3.0.2 (https://www.audacityteam.org). In the R programming environment (R Core Team 2013), we used the functions autodetect, specan, and segment from R packages warbleR (Araya-Salas and Smith-Vidaurre 2017) and soundgen (Anikin 2019) on the annotated recordings to impose a frequency bandpass filter of 0 to 6 kHz and extract 7 acoustic parameters previously established as important for female preference and species recognition in *D. cinereus* (following the methods described in Laird et al. 2016): mean dominant frequency for both high and low frequency bands (kHz), call duration (ms), call rate (calls/sec), pulse number (per call), pulse rate (pulses/ms), and pulse duration (ms). Fundamental frequency, frequency peaks, mean frequency, relative amplitude (loudness), number of calls per minute for each continuous effort (i.e., separate efforts separated by at least 30 s of rest), number of territorial calls, and presence of nearby males (within 1 m of the calling male) were also recorded for each male.

### Two-choice playback experiments

Two-choice playback experiments were conducted in the field over 2 summers (2020–2021) using a ∼2.9 square meter mesh arena placed on the pond shoreline at the same pond where calls were recorded (Sansbug mesh tent, model SB-2P-TF-GT). Setup of the arena followed a standard 2-way playback experimental design (Gerhardt 1995) (Figure 1A). Briefly, 2 field speakers (SME-AFS portable field speakers, Saul Mineroff Electronics, Inc.) were placed on either end of the arena exactly 2 m apart and oriented at a 45° angle toward the center-front of the arena. One meter from the center point between the speakers in the direction of the opening, a spot was marked with tape as the release point for each female so that speakers were oriented in her direction, and she was equidistant from each speaker. Identical 6 cm by 6 cm cut-out color photographs of a calling male Green Treefrog were attached to each speaker to provide a visual stimulus in addition to the auditory signals, since multimodal signals may be more effective at eliciting female responses in treefrogs without biasing choice (Laird et al. 2016; Zhu et al. 2021; Augusto-Alves et al. 2025).

**Figure 1:**
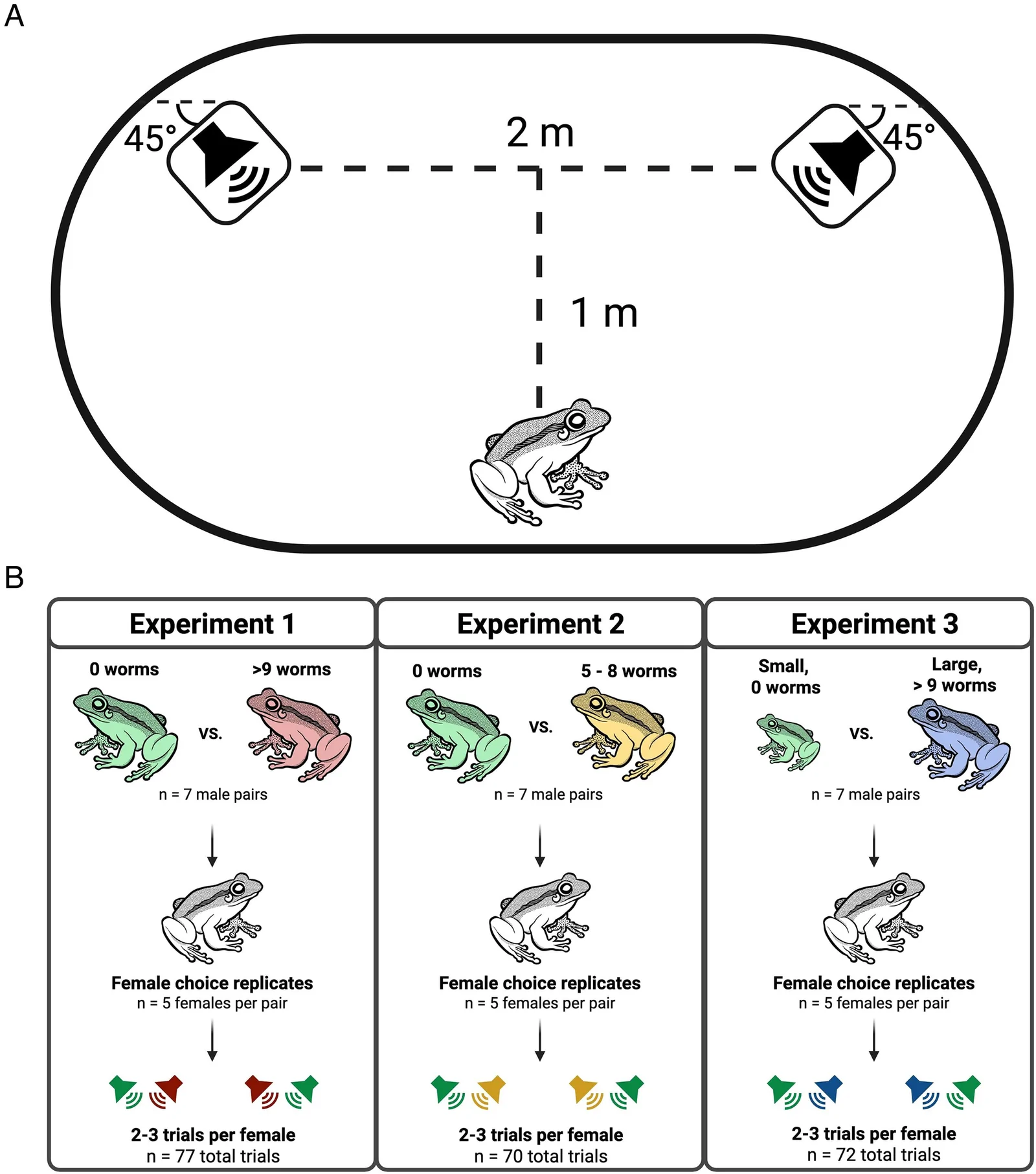
Experimental setup and design. (A) Two speakers were placed at 45-degree angles on either side of an arena with 2 m of distance between them. Females were placed at a release point 1 m below the midpoint between the 2 speakers. (B) Seven unique pairs of male calls were used for each of the 3 experiments, which were each tested on 5 separate females spread across 2 breeding seasons (for a total of at least 35 replicates per experiment). Each female was tested twice on each pair with opposite orientations of right and left speakers, with a third tie-breaker trial if outcomes from the first 2 trials were conflicting. Created in BioRender. Goodnight, S. (2026) https://BioRender.com/7eul761.

Receptive female frogs were collected by hand by capturing frogs in amplexus on evenings after rain events during an active chorus (Ryan and Keddy-Hector 1992; Gerhardt 1995). Females were separated from the males for use in the 2-choice playback experiments. After a 10-minute rest period after separation from the male, females were placed in the arena one at a time at the marked release point, and calls were played simultaneously from both speakers. Calls playing from each speaker were standardized to 90 decibels by adjusting the volume and measuring the output using the DecibelX application for iPhone (Skypaw Co. 2020) (Murphy and King 2016). A choice was recorded when the female stayed within 15 cm of a speaker for 60 continuous seconds, after which the trial was concluded, time-to-choice was recorded, and she was removed from the arena. Each female was continuously observed in the arena for up to 10 min per trial. If no choice was observed, then the female was removed from the arena at the 10-min mark. Between trials, the playbacks were discontinued for a 10-min “cool down” period for the female, after which the calls coming from the speakers were flipped in orientation—that is, the call playing from the right speaker in the previous trial was now playing from the left speaker, and vice versa. After 10 min the same female was then tested with the same combination of calls, but now flipped in orientation, a second time. If at the conclusion of the second trial, a female selected a different call than her first trial (or did not choose either), a third “tie-breaker” trial was performed following the same methods with a now randomized speaker orientation. The loudness of the calls was always reset to 90 decibels for both speakers before each trial. Female body size (mass in grams and SVL in mm) and infection abundance (number of worms visible in the mouth) were collected for each female in the same manner as the recorded males only after all her trials had been completed, after which all females were re-released at the original site of capture.

To determine whether tongueworm parasite infection in male signalers influenced female choice, we aggregated calls into 3 infection categories: uninfected, moderately infected (5–8 adult worms), and heavily infected (>9 adult worms). We chose these categories based on top 20% of infection abundances in this population, half of which had 5–8 parasites and half had >9 parasites (median = 13; mean = 13.8; max = 24). We conducted 3 separate 2-choice playback experiments designed to evaluate female choice of 3 different pairwise comparisons (Figure 1B). For Experiment 1, we tested female choice of calls from heavily infected males against calls from size-matched uninfected males, to test whether females preferred calls from uninfected males. For Experiment 2, we tested calls from moderately infected males (5–8 adult worms) against size-matched uninfected males to determine if magnitude of infection influenced female preferences. Finally, for Experiment 3 we tested calls from large, heavily infected males (>9 adult worms) against calls from smaller, uninfected males to test any possible preference for larger males (Gerhardt et al. 1987; Asquith and Altig 1990) against potential avoidance of parasitized males. For each experiment, 7 unique pairs of recordings were played for 5 different female frogs each, for a total of 35 playbacks. Females were re-used for multiple recording pairs, but were rarely (*n* = 2) re-tested on 2 different pairs in the same Experiment. Each of the 35 playbacks included 2 or 3 trials (where the calls were switched between speakers) for each female and each replicate pair for a total of 219 trials across all 3 experiments (Figure 1B).

For Experiment 1 and Experiment 2, all call recordings were from size-matched male Green Treefrogs. That is, the calls of highly or moderately infected males used in both experiments were matched with calls from uninfected males with a similar mass (<0.01–0.35 g of difference in body mass [mean difference in body mass per pair ± standard deviation = 0.124 g ± 0.121; mean body mass per recorded male ± SD = 5.492 g ± 1.282]). We also ensured that the calls from matched infected and uninfected males were recorded on the same date in the same weather and temperature conditions to the extent possible, to avoid confounding impacts of environmental conditions on call characteristics (Gerhardt 1978). When we did not have recordings from size-matched males that were also recorded on the same date, we used a mass- and temperature-matched recording taken on a different date (*n* = 8 across both experiments). For Experiment 3, which tested the relative importance of male body size against parasite abundance, we paired the calls of males recorded from the same population on the same night or in similar environmental conditions. The large, infected males in these experiments were between 1.2 and 3.3 g larger (mean difference in body mass per pair ± SD = 2.17 ± 0.69 grams) than the small, uninfected males to ensure that size-based call metrics would differ. We tested whether slight differences in temperature at time of recording, temperature at time of playback trial, mass differences between the competitors, and infection abundance in the female affected female choice in our statistical analyses.

Because we needed to select paired calls based on size and environmental conditions to limit bias, calls from some infected males were re-used in multiple playback trials and thus competed with more than one competitor. We account for the potential influence of this nonindependence in all statistical analyses (detailed below).

### Statistical analyses

All statistical analyses were performed using the R programming environment. We used the packages glmmTMB (Magnusson et al. 2017) and brms (Bürkner 2017) to fit generalized linear models (GLMs) or generalized linear mixed models (GLMMs) and Bayesian mixed models (BRMs) respectively. For GLMs and GLMMs we used DHARMa (Hartig and Hartig 2017; Hartig 2020) and the bbmle (Bolker and Bolker 2017) packages for model interrogation and model comparisons. We used trace plots and Gelman-Rubin R-hat statistics to evaluate convergence of Bayesian mixed models and the loo package (Vehtari et al. 2017) to perform leave-one-out cross validations. The ggeffects (Lüdecke 2018) package was used for generating marginal estimates and model predictions for all analyses, and ggplot2 (Wickham 2011) for visualization of predicted relationships.

To investigate whether male body size (SVL in mm) or time of recording (month; May-August) predicted parasite infection abundance (i.e., number of adult parasites per individual), we compared GLMs including 4 different fixed effects structures: 1) log-transformed SVL (lnSVL), 2) month, 3) the additive effects of lnSVL and month, and 4) an intercept-only null model. Furthermore, we fit models with each of these 4 fixed effects structures assuming 4 different count or overdispersed-count distributions: Poisson, zero-inflated Poisson, and 2 forms (allowing variance to increase linearly or quadratically with the mean) of negative binomial and zero-inflated negative binomial distributions. Model selection was based on lowest sample size-corrected Akaike's Information Criterion (AICc) score (Anderson et al. 1998).

To test whether parasite abundance was correlated with frog body condition, we calculated 2 different indices for male body condition. The scaled mass index is calculated as $M=M_{i}\;{\left[\frac{L_{0}}{L_{i}}\right]}^{b}$ where *M_i_* is the body mass of the individual, *L*_0_ is the mean SVL of the population, *L_i_* is the SVL of the individual, and *b* is a scaling component estimated using a log-linear regression of mass vs. SVL (see Peig and Green 2009 for a detailed explanation of this method). The ratio-based index of body condition is calculated by dividing body mass (in grams) by SVL (in mm) (Labocha et al. 2014). For each condition index, we fit GLMs with Gaussian error distributions and log link functions to test whether condition index varied as a function of infection abundance of adult worms.

#### Call recordings

Out of 199 total recordings, 22 calls were removed before analysis due to insufficient recording length, poor recording quality, or excessive background noise. To test whether parasite abundance affected the characteristics of 7 key call parameters known to be important for mate attraction and species identification in this species (call duration, high and low mean dominant frequency, pulse duration, pulse rate, pulse number, and call rate) we fit Bayesian mixed-effects models with default uninformed priors (Bürkner 2017) and skew-normal error distributions to each call parameter individually. Because we were interested in determining how infection abundance affected call characteristics, we first analyzed all the data (infected and uninfected frogs *n* = 177) and then ran a second analysis focused only on the recordings from infected frogs (*n* = 72). We included individual frog ID as a random effect in all models, except for call rate since there was only observation of this summary variable per individual (in calls/sec), to account for the fact that we extracted data from multiple calls per frog. We included SVL as an offset in each model to size-adjust call parameters to remove known covariation with body size (Gerhardt 1987; Laird et al. 2016). The relationships between SVL or body mass and each call parameter are presented in Supplementary Figures S1 and S2. We used Gelman-Rubin statistics and traceplots to ensure models converged and to evaluate model performance for each analysis.

#### Two-choice playback experiments

For the 2-choice playback response experiments, 6 total trials across the 3 experiments that used the same female (ID# PF21) were removed before performing any statistical analysis because she was non-responsive to the stimuli. Thirty-one additional trials were removed prior to analysis because the females did not choose either speaker within the time limit (*n* = 13 for Experiment 1, *n* = 10 for Experiment 2, and *n* = 8 for Experiment 3). Females were re-tested on more than one unique experimental pair of calls, but generally not tested more than 3 times total, resulting in a total number of *n* = 41 females tested against unique pair combinations across the 3 Experiments.

To analyze female choice, we took 2 separate approaches. First, we tested for differences in female choice for one or the other recorded male advertisement call in each of the three 2-choice playback experiments using Bayesian mixed models with default uninformed priors (Bürkner 2017). For each analysis we fit Bernoulli distributions (i.e., binary choice data), where a choice of the uninfected male in each experiment was modeled as a 1 while a choice of the infected male was modeled as a 0. We included female ID within pair ID as nested random effects for all models to account for the non-independence between the repeated trials that were conducted for each female (due to speaker switching). The pair ID used in the nested random effect also accounted for non-independence of pairs that were tested with the same male call, but against different competitors. We again used Gelman-Rubin statistics and traceplots to ensure convergence and interrogate model fits.

Second, to gain a more comprehensive assessment of associations between call characteristics, parasite infection abundance, and mate choice we tested whether 3 important call parameters (call duration, rate, and frequency) from the calls used in the three 2-choice playback experiments predicted female choice when one competitor was infected. We focus on call duration, call rate, and dominant frequencies because these call characteristics likely predict female preference more than pulse characteristics (Gerhardt 1978, 1987, 1991; Leary et al. 2021). For each experiment, we fit GLMMs that modeled the proportion of trials that each male was chosen as a function of 21 different combinations of predictor variables (call duration, call frequency [both low and high dominant frequencies], call rate, and infection abundance—see Supplementary Tables S4 through S6 for a list of all models). All models assumed binomial error distributions and logit link functions. Pair ID was again included as a random effect in all models to account for non-independence between experimental pairs. We also did an additional analysis testing whether call duration, call frequencies, call rate, and body size (for the first 2 experiments only) differed between uninfected and infected males from each of the 3 experiments using log-normal GLMMs with pair ID as a random effect. Results from these models are summarized in Table 1. Model selection was performed assuming the model with the lowest AICc score was the most likely. However, when multiple models were ≤2 ΔAICc units from the top, we selected the most parsimonious (fewest parameters) model.

**Table 1:** Estimated marginal means and 95% confidence intervals for differences in frequencies and call duration of uninfected and infected males used in playback experiments.

	Dominant frequency (high band, kHz)		Dominant frequency (low band, kHz)		Call duration (ms)	
	Infected	Uninfected	Infected	Uninfected	Infected	Uninfected
Experiment 1	2.98*, 2.87–3.11	3.17*, 3.04–3.31	0.999, 0.948–1.05	1.01, 0.939–1.071	129*, 117–142	144*, 131–159
Experiment 2	3.09, 2.97–3.21	3.03, 2.79–3.27	0.922*, 0.881–0.965	0.980*, 0.936–1.026	155*, 144–167	144*, 133–155
Experiment 3	3.49*, 3.33–3.65	2.97*, 2.84–3.11	0.999*, 0.967–1.030	1.074*, 1.039–1.110	142, 121–162	147, 134–159

Values where there were significant differences between the infected and uninfected male groups in the given experiment are marked with asterisks. There were no differences in call rates between uninfected and infected males in any of the 3 experiments.

## Results

Among the 4 best models for tongueworm infection abundance, all provided equivalent fits to the data (ΔAICc < 2). Each model included body size (SVL), while 2 also included month as a predictor (Supplementary Table S1). The remaining difference among the top models were that 2 assumed linear growth (one per predictor set), and 2 assumed quadratic growth of the variance relative to the mean within a zero-inflated negative binomial framework. Although the SVL-only model is more parsimonious, we interpret the top model (ΔAICc = 0) that includes both SVL and month, since frogs arriving earlier in the season are expected to have a lower chance of infection given the natural history of the system. The number of worms increased with male SVL (slope = 3.11 [95% CI = 0.48–5.75]) (Figure 2) and all males had more worms on average in later months (predicted number of parasites for August = 3.86 [2.45–6.09] and July = 5.06 [3.44–7.46]) than earlier months (predicted number of parasites for May = 0.58 [0.25–1.32] and June = 3.00 [1.36–6.63]) (Figure 2). Interestingly, body condition, calculated using either a scaled mass index (intercept = 1.68 [95% CI = 1.65–1.72]) or ratio-based index of condition (intercept = −0.25 [−0.28 to −0.22]), did not correlate with tongueworm infection abundance.

**Figure 2:**
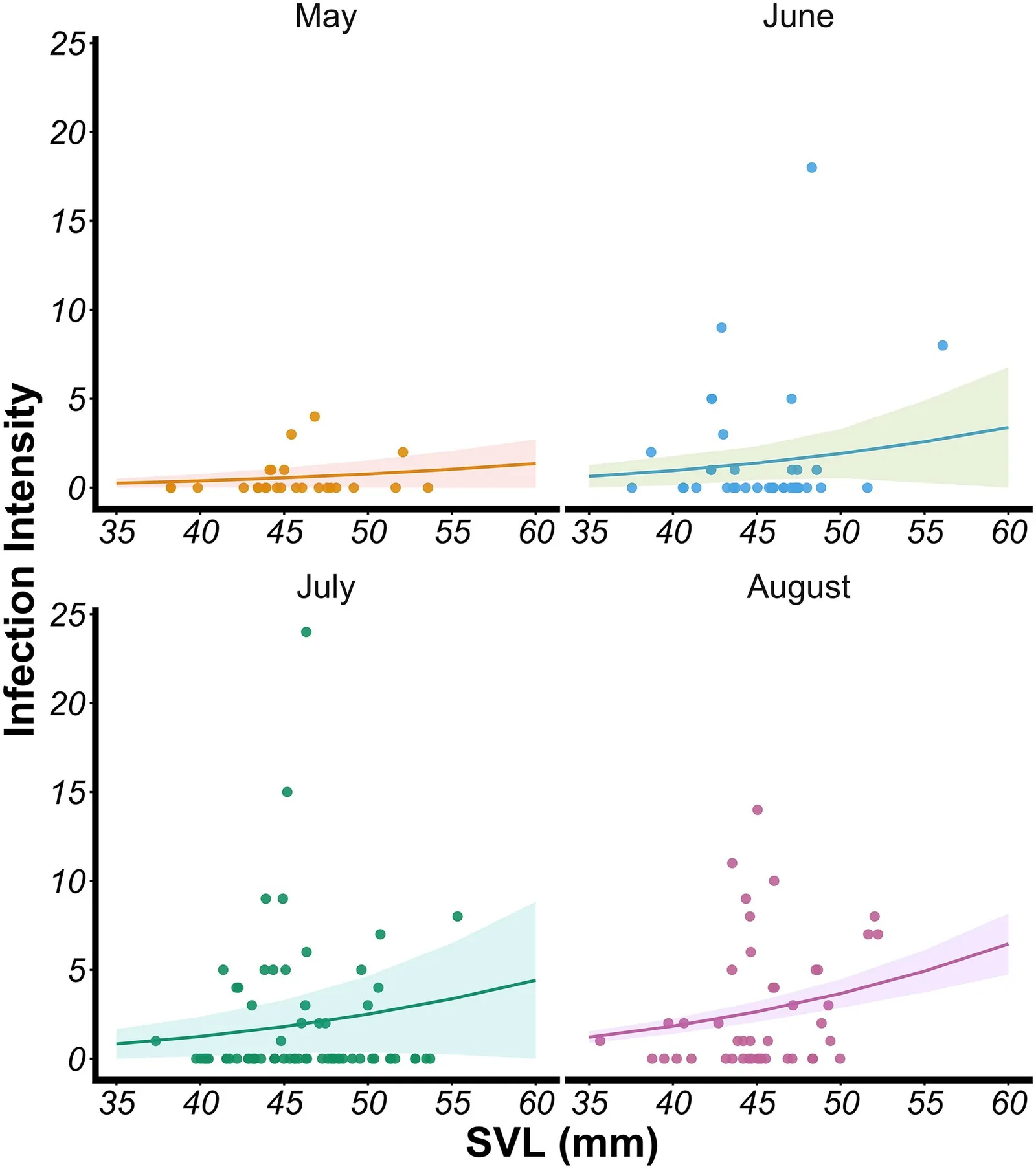
Relationship between body size (SVL in mm) and tongueworm infection abundance in male frogs recorded May–August. The fitted lines are predicted relationships from a zero-inflated generalized linear model (GLM) with confidence envelopes, plotted over raw data from frogs recorded each month.

### Call recordings

Analyses of call characteristics in both the entire population (infected and uninfected frogs) and the subset of infected frogs yielded qualitatively similar results (Figures 3 and 4, Supplementary Tables S2 and S3). Therefore, we focus our discussion on infected frogs. Uninfected frogs may include healthy, unparasitized males in good condition, as well as unparasitized males in poor condition due to factors other than infection (and those that are unparasitized because they were non-foraging). Although for a field-based study our sample size overall was large, there were many more uninfected males than males with any amount of infection abundance (*n* = 105 uninfected vs. *n* = 72 infected), leading to intractably large amounts of variation in the uninfected frogs that made inference of the full dataset difficult. Additionally, even infected individuals often had very few worms (<3) which led to a highly skewed distribution of our predictor variable of infection abundance, an issue unable to be ameliorated in our statistical framework when all frogs were included in analyses. Coefficient estimates and confidence intervals for the analysis of the full dataset are summarized in Supplementary Table S2. Among infected frogs, tongueworm infection abundance was negatively correlated with high (estimate = −0.048 [95% CI = −0.08 to −0.01]) and low (−0.041 [−0.07 to −0.004]) dominant call frequencies (Figure 3B, C), call duration (−0.004 [−0.01 to 0.00]) (Figure 3A), pulse number per call (−0.080 [−0.14 to −0.02]) (Figure 4A), and pulse rate (−0.068 [−0.11 to −0.03]) (Figure 4B). We found little effect of tongueworm parasites on call rate (−0.029 [−0.06 to 0.002]) (Figure 4D) or pulse duration (0.003 [−0.02 to 0.24]) (Figure 4C).

**Figure 3:**
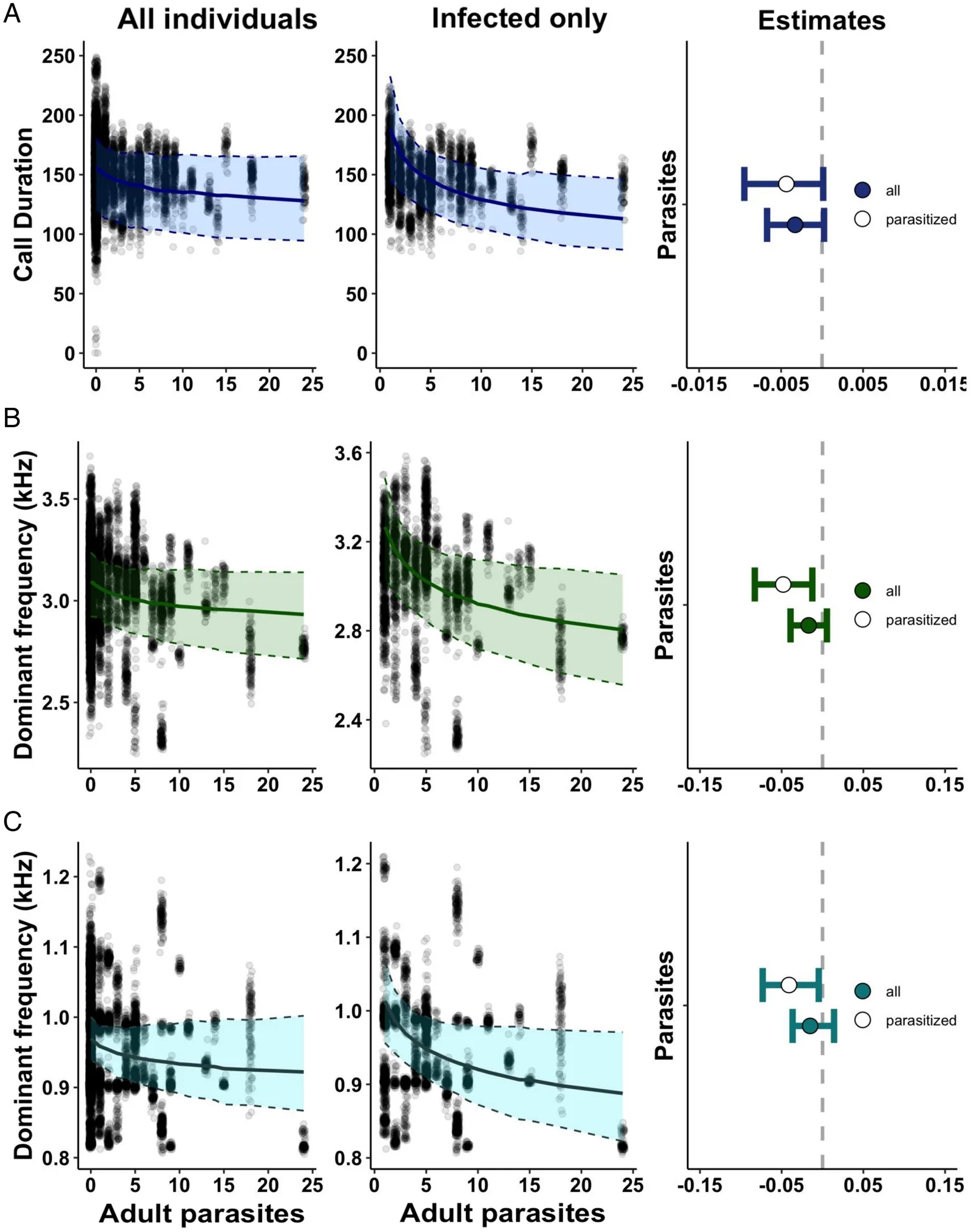
Effects of tongueworm parasite infection on call duration (top row), dominant frequency (high band, middle row), and dominant frequency (low band, bottom row) for all frogs (*n* = 177) and infected frogs only (*n* = 72). Predicted values (colored lines) are plotted over raw data on the left of each panel, and error envelopes are 95% credible intervals. Coefficient estimates from each model for parasite abundance are on the log scale in the rightmost column. Error bars are also 95% credible intervals.

**Figure 4:**
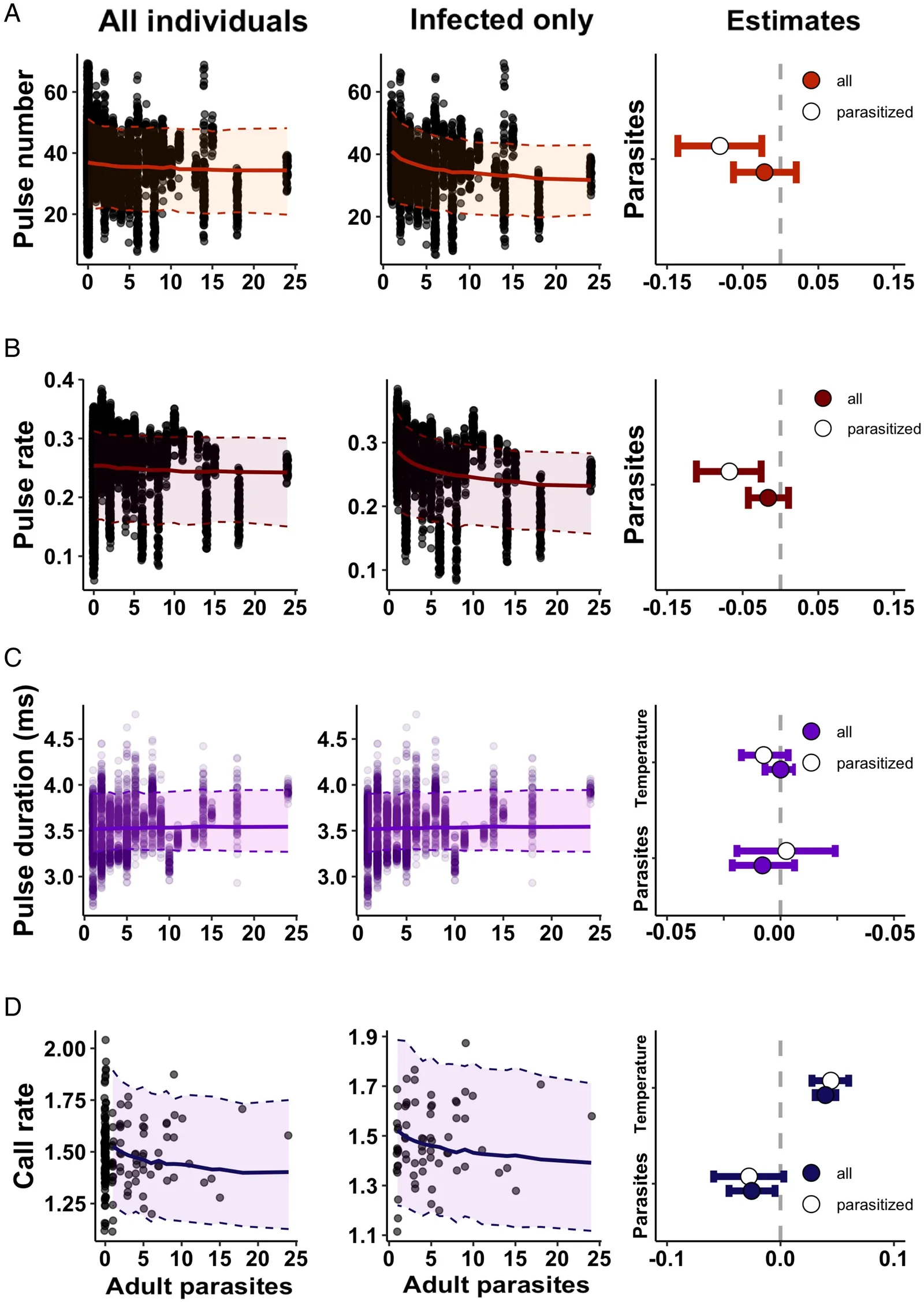
Effects of tongueworm parasite infection on pulse characteristics and call rate for all frogs (*n* = 177) and infected frogs only (*n* = 72). Predicted values (colored lines) are plotted over raw data on the left of each panel, and error envelopes are 95% credible intervals. Coefficient estimates from each model for parasite abundance are on the log scale in the rightmost column. Error bars are also 95% credible intervals. Plotted lines for effects of temperature where applicable are excluded here but are provided in Supplementary Figure S1.

For pulse duration and call rate, temperature was an additional predictor (Supplementary Table S3, Supplementary Figure S3). Specifically, temperature was slightly negatively correlated with pulse duration (−0.010 [−0.02 to −0.003]) but positively correlated with call rate (0.044 [0.03–0.06]). There was no apparent effect of barometric pressure on any call characteristics we measured. Coefficient estimates and associated confidence intervals for both parasite abundance and temperature for each call characteristic are summarized in Supplementary Table S3.

### Two-choice playback experiments

Our Bayesian models revealed distinct female preferences across the 3 experiments. In Experiment 1 (heavily infected vs. uninfected male calls) females strongly preferred the uninfected male call, choosing them over 3 times more frequently than heavily infected males (probability of choosing the uninfected call = 0.87 [95% CI = 0.59–0.96]; Figure 5). Surprisingly, this preference reversed in Experiment 2 (moderately infected vs uninfected), where females selected moderately infected calls nearly twice as often as uninfected calls (= 0.14 [0.19–0.45]) (Figure 5). In Experiment 3 (small uninfected vs. large heavily infected), females showed no preference, choosing both options equally (probability = 0.50 [0.13–0.88]) (Figure 5). Notably, neither tongueworm burden nor environmental conditions significantly influenced female decision latency in any experiment. Furthermore, female choice patterns were not predicted by environmental factors, female infection status, or body size differences between competing males in any 2-choice playback experiments.

**Figure 5:**
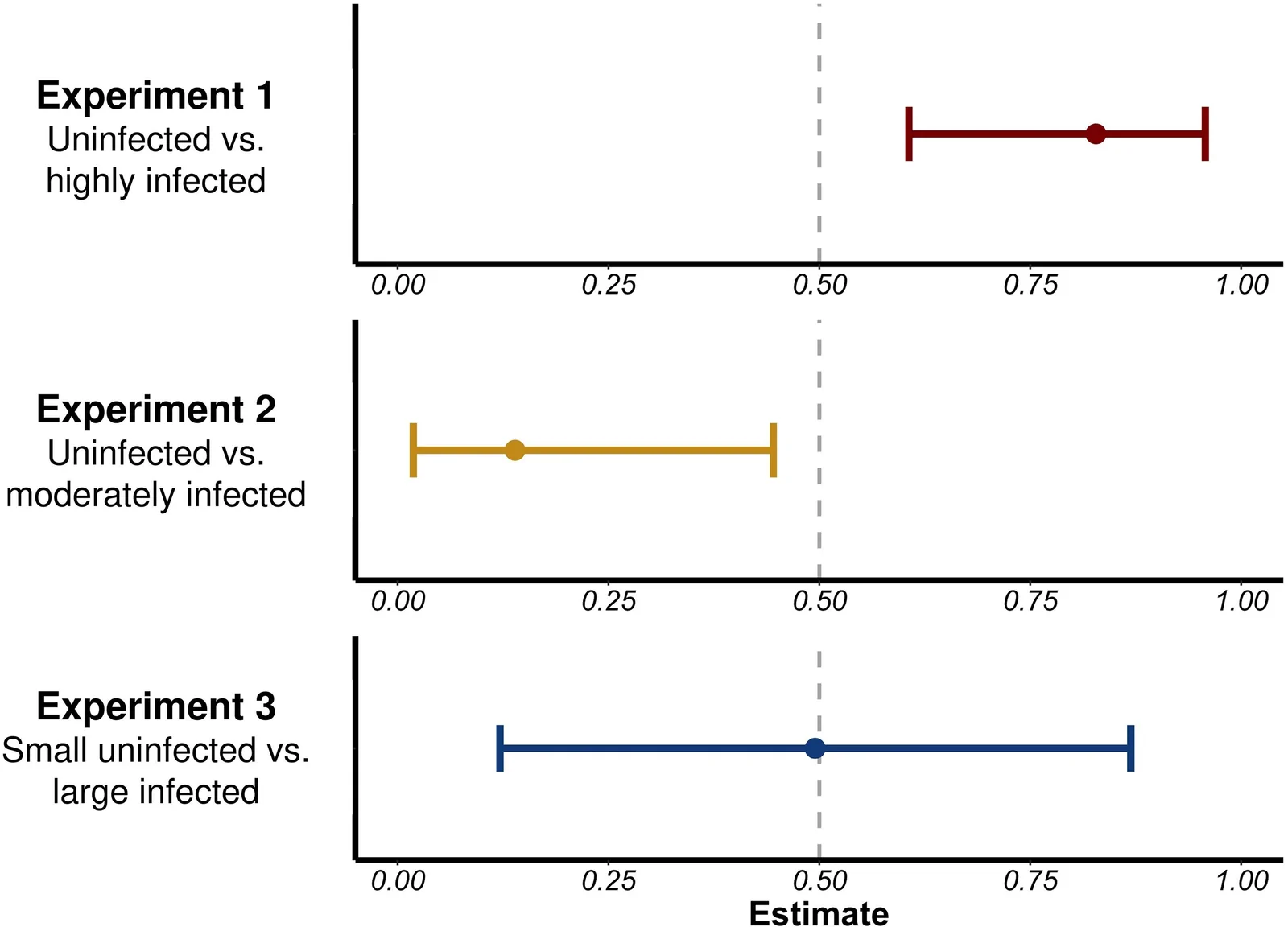
Intercepts for each of the three 2-choice playback experiments. Error bars represent 95% credible intervals. Intercepts to the right of the dashed *y* = 0.5 line indicate that the call recorded from uninfected males were selected more often than calls recorded from males with parasite infections, while estimates to the left of the line indicate the infected male call was selected more often.

To investigate how call parameters interacted with parasite infection to drive female choice, we analyzed the calls from males used in the 2-choice playback experiments (*n* = 13 for Experiment 1, *n* = 13 for Experiment 2, and *n* = 10 for Experiment 3) for our second approach using GLMMs. In Experiment 1, there were 5 models that provided equivalent fits to the data (ΔAIC≤1.9). Here, we interpret the top and most parsimonious model (fewest parameters), which included the additive effects of number of tongueworms and dominant frequency (high band). These 2 variables appeared in 4 of the 5 top models (Supplementary Table S4). We found that the probability of a female selecting a call increased with high dominant frequency (5.36 [−0.32 to 11.04]) (Figure 6A), and decreased with number of tongueworms (−0.100 [−0.18 to −0.02]) (Supplementary Figure S4A).

**Figure 6:**
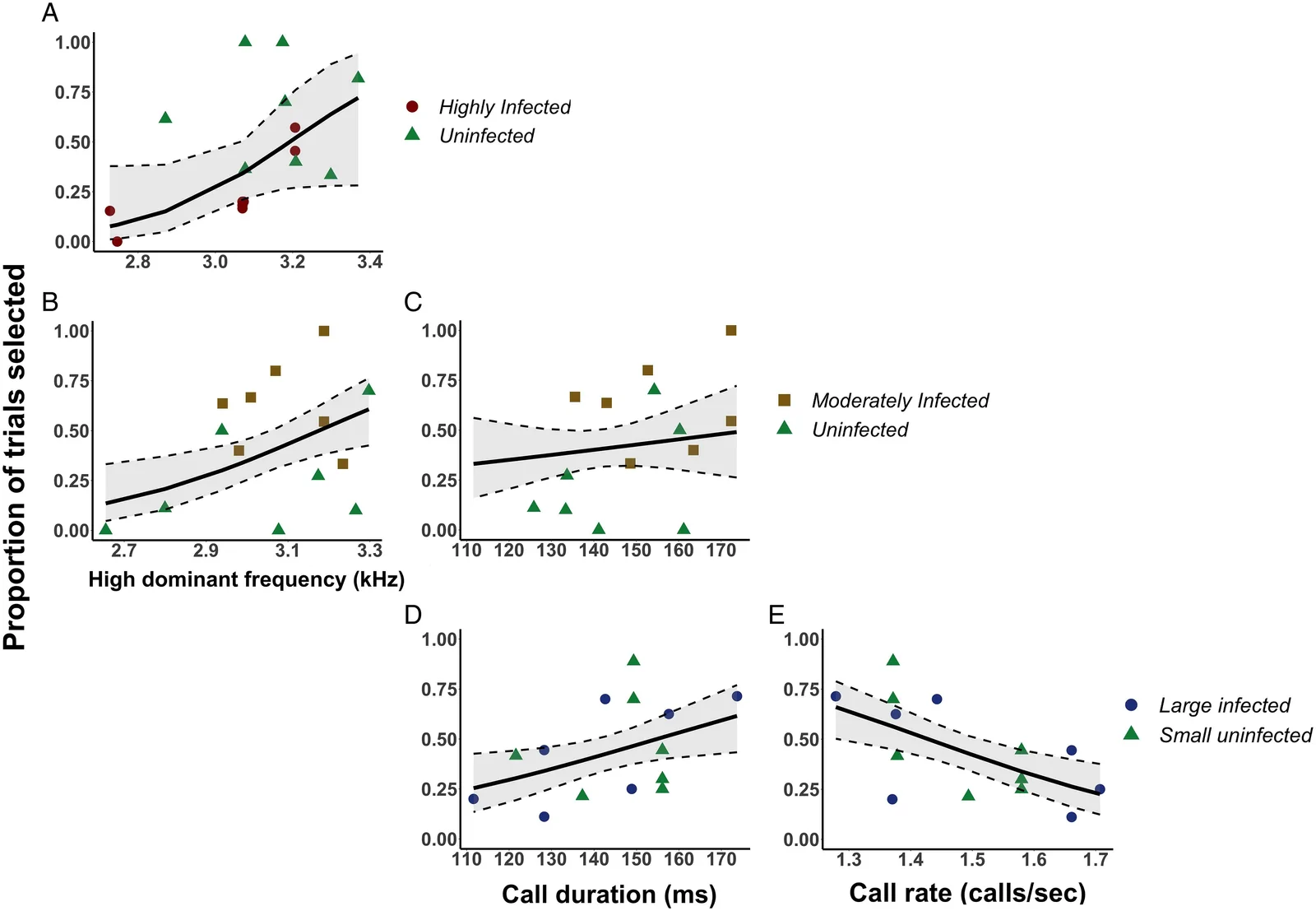
Relationships between call frequency, duration, rate, and female choice for playback Experiment 1 (A), Experiment 2 (B, C), and Experiment 3 (D, E). Plotted lines are predicted relationships from binomial generalized linear mixed models and error envelopes are 95% confidence intervals. The y-axis is the proportion of trials each male call was selected over the competitor for each experiment. Only call characteristics that correlated with female choice, based on the top model for each experiment, are visualized here.

Similar to Experiment 1, analyses of the data from Experiment 2 yielded 3 models that provided equivalent fits to the data (ΔAIC≤1.9). All 3 models included tongueworm infection abundance, dominant frequency (high band), and call duration (ms); so, we interpret the top model for our results, which included infection abundance and the interaction between call duration and frequency (Supplementary Table S5). The probability of selecting a call in Experiment 2 increased with dominant frequency (2.37 [−0.01 to 4.76]) (Figure 6B) and number of tongueworms (0.182 [0.06 to 0.30]) (Supplementary Figure S4B) but decreased with call duration (−6.72 [−14.34 to −0.90]) (Figure 6C). The interaction estimate between call duration and frequency was 2.30 (−0.19 to 4.8).

Following our previous analysis, the top model for Experiment 3 did not include the number of tongueworms nor call frequency variables. Instead, in this experiment, mate choice decisions appeared to be driven by call rate and call duration (Supplementary Table S6). Specifically, the probability that a call was chosen declined with call rate (−4.43 [−7.21 to −1.65]) and increased with call duration (0.42 [0.06 to 0.78]) (Figure 6D, E).

## Discussion

The Hamilton–Zuk parasite sexual selection hypothesis states that the fitness impacts of parasites constrain the expression of secondary sexual traits in infected hosts, and the resulting honest signals of condition influence mate choice decisions (Hamilton and Zuk 1982; Read 1987; Zuk 1992; Molnár et al. 2013). In this study, we show that tongueworm parasite infection in Green Treefrogs affects the expression of secondary sexual call traits, but changes in male call characteristics were nuanced, and their impact on female choice complex. We show that the basic expectations of the Hamilton–Zuk hypothesis may not hold for trophically transmitted parasites such as digenean trematodes that tend to accumulate in hosts that are larger, older, or more fit (i.e., the most active foragers, most aggressive males, or the most experienced individuals), due to the correlation between prey consumption over time and infection abundance (Connors et al. 2010; Comas et al. 2014; Munson et al. 2020).

### Impacts of parasite infection on advertisement call characteristics

In this study, larger male body sizes were correlated with higher tongueworm infection abundances; larger males consume more prey and so are expected to have higher condition as well higher infection rates of trophically transmitted parasites like tongueworms (Hall et al. 2007; Sanchez et al. 2018), although we found no detectable negative effects of infection on body condition per se. Tongueworm infection abundance was also associated with changes in male advertisement calls that were contrary, in part, to Hamilton–Zuk expectations. We resolved a negative relationship between tongueworm infection abundance and call dominant frequencies (in kHz) even after controlling for body size. Since prior studies have documented female preference for larger males with lower frequency calls in multiple frog species (Howard et al. 1998; Hunt et al. 2009), including Green Treefrogs (Gerhardt 1978; Asquith and Altig 1990; Laird et al. 2016), our results suggest that choosy females may face a distinct conflict between choosing “preferred” male call traits like lower frequencies, and avoiding traits such as larger body sizes that might indicate a higher likelihood that a mate is parasitized. However, advertisement call duration (in ms) was negatively affected by parasite abundance in the caller, and studies have shown that calls of longer duration may be preferred by females in some contexts, especially when the range of durations available for selection falls within realistic boundaries (Gerhardt 1987; Leary et al. 2021). This trait is likely selected for via mate choice decisions because it positively correlates with male size, health and condition, or energetic effort (Gerhardt 1987; Höbel and Gerhardt 2003; Leary et al. 2021; Supplementary Figures S1 and S2). Therefore, in contrast to our findings with call frequency, longer call durations can signal both low parasite infection abundance and better condition of the signaler.

Contrary to Hamilton–Zuk expectations, we observed no clear effects of parasite infection on male body condition or apparent health; however, we note that we cannot rule out that high infection abundances of tongueworms impacted male health in other ways that we did not measure (e.g., Moretti et al. 2014). For instance, parasites have been shown to increase stress hormone levels in vertebrate hosts (O’Dwyer et al. 2020). Leary et al. 2021 showed that Green Treefrogs with higher stress hormone (corticosterone) levels displayed shorter calls and lower vocal effort, introducing one potential mechanism for why infected males had shorter calls in our study. The association between reduced call duration and tongueworm infection could also stem from the accumulation of adult worms in the mouth and throat of heavily infected males, and thus restricted airflow to the vocal sac. Physical blockage of respiratory or auditory passages by parasites in other species is known to impair the production (Cade and Wyatt 1984; Kleindorfer et al. 2019) as well as the reception (Ferry et al. 2011; Bevier and Gorman Gelder 2018; Reichert et al. 2023) of vocalizations. Interestingly, the sister species to the tongueworm, the “earworm” *H. eccentricus*, lives in the eustachian tubes of frog hosts and can impact host hearing ability (Bevier and Gorman Gelder 2018). Thus, we see that this genus of parasite specializing in microhabitats that govern host acoustic signaling may have impacts on amphibian mate choice by manipulating communication between both the signaler and receiver. Outside of the current study, there is little, if any, data available on parasites or other deleterious conditions physically impairing breathing and airflow in anurans and impacting signal production; therefore, our study highlights additional questions for novel work exploring the specific mechanisms of observed effects.

Finally, female Green Treefrogs prefer higher call rates since this trait may be a signal for healthier males in better condition that can generate more calls over time (Gerhardt 1991). However, we found no strong effects of infection on male condition and surprisingly only a mild negative effect on call rate in terms of the number of calls produced per unit time. The only robust trend we observed was a positive relationship between ambient temperature and call rate, which is consistent with similar studies recording male Green Treefrogs and other anuran species in the wild (Asquith et al. 1988; Höbel and Gerhardt 2003; Ziegler et al. 2016). Since increasing call rate is energetically costly for signaling males, especially absent a compensatory reduction in call duration (Gerhardt 1994b; Ziegler et al. 2016), higher temperatures increase metabolic rate and allow these endothermic animals to maintain the energy levels to call at higher rates for longer periods (Gillooly et al. 2001; Ziegler et al. 2016). Furthermore, larger males on warm nights are likely foraging more to compensate for increased energy needs—enhancing infection risk by trophically transmitted parasites like tongueworms. Although we resolved little direct impact of parasites on call rate in this study, future work is needed to evaluate how parasite infection impacts call characteristics across a range of environmental conditions.

Because frog advertisement calls are multimodal and transmit complex information about the caller's size, health, and species identity through diverse concurrent acoustic components, changes in the covariance structure of some call characteristics rather than static single traits (e.g., duration vs. frequency) are important for female choice. We found moderate differences in the pulse structure of male calls associated with parasite infection abundance, where highly infected males had lower numbers of pulses per call and lower pulse rates. Although the relationship between call pulse structure and female preference is more ambiguous that that of other traits like frequency, pulse rate, duration, and number are known to be important for species recognition in treefrogs (Gerhardt 1991). For instance, female Green Treefrogs have shown preference for a relatively narrow range of pulse rates and discriminate against calls that have too low or too high values (Gerhardt 1982). Additionally, higher numbers of pulses are generally positively correlated with call duration, and call duration can be predictive of female preference (Gerhardt 1987). Indeed, pulse numbers of our recorded male calls decreased with increasing infection load similar to call duration. Moreover, there was a slight negative effect of temperature on pulse duration, where calls recorded at higher ambient temperatures had slightly lower pulse durations. Parasite-altered call pulse structure may have impacts on female recognition of the signal as well as perceived mate quality, and our study raises questions for future research on the mechanisms behind parasite-mediated female responses to the structural features of advertisement calls.

### Parasite-mediated mate choice patterns

Our findings from the recorded advertisement call analyses offer compelling questions about how parasites influence complex vocal signals, and how choosy females respond to these signals. In our 2-choice playback experiments, we found that mate choice by female Green Treefrogs did not follow expected patterns when the males had tongueworm parasite infections. Overall, females did not select the calls of the most highly infected males (>9 parasites) in 2-choice playbacks when they were competed with calls from size-matched uninfected males, but preferred the calls of moderately infected males (5–8 parasites) over those from uninfected males. This nonlinear response to parasite infection does not conform to traditional Hamilton–Zuk expectations, and highlights the need for more mechanistic research exploring how parasites that infect via trophic transfer modulate female preference.

Specifically, in both playback experiments where the calls of size-matched males were presented to receptive females (Experiments 1 and 2), females consistently chose calls with higher dominant frequencies over calls with lower dominant frequencies, regardless of whether those calls came from infected or uninfected males. This directly contrasts with prior studies on *D. cinereus,* where females consistently chose natural calls with lower dominant frequencies over natural calls with higher frequencies (Gerhardt 1978; Gerhardt et al. 1987; Laird et al. 2016). These results suggest that parasite infection status is an important factor in mate choice; while lower dominant frequency may positively correlate with preferred male traits, such as body size and condition, it also may be an indicator of high parasite infection. Thus, females in parasitized populations may prefer call characteristics that reduce the risk of selecting parasitized mates. Further supporting this hypothesis, in Experiment 3, when females were presented with calls recorded from large males vs. small males, we did not observe clear preferences—although we did not directly test female preference for large males in the absence of parasites, this tentatively suggests that heavy parasite infections in larger males may compete with the commonly observed female preference for call characteristics that signal large male body size (Gerhardt 1978; Asquith and Altig 1990).

Importantly, sexual selection for dominant frequency in Green Treefrog calls has often been characterized as stabilizing rather than strictly directional, with experimental studies using synthesized calls showing that females generally prefer calls within a relatively narrow range of species-specific values rather than the absolute lowest possible frequencies (Gerhardt 1991; Gerhardt and Huber 2002). Although dominant frequencies do often correlate with male body size, lower-frequency calling does not necessarily translate into increased mating success (see Gerhardt et al. 1987). Thus, our results should not be interpreted as evidence that parasite infection in a calling male necessarily reverses or weakens directional selection, but that parasites can influence how choosy females weigh frequency relative to other call traits such as call rate and duration.

We observed that males with higher call rates were selected less often by females, which was also antithetical to expectations, as larger, healthier males are able to produce more calls per unit time, which is typically a preferred trait by females in this species (Gerhardt 1978; Leary et al. 2021). Interestingly, although the impact of parasites on call rate were statistically ambiguous, parasite infection was actually slightly negatively correlated with this call trait. Regardless, this population of female Green Treefrogs made mate choice decisions that were not consistent with expectations from earlier studies on this species nor with Hamilton–Zuk theory more generally (Gerhardt 1978; Asquith and Altig 1990; Leary et al 2021). Therefore, our results suggest that infection by trophically transmitted tongueworm parasites disrupts some expected patterns in mate choice decisions. The non-selection of infected males with favorable call traits like low dominant frequency and higher call rate may mean that parasites alter other aspects of male calls that affects how females interpret signals and evaluate male size or perceived quality.

We observed a positive trend between the probability of a call being chosen by a female and call duration in both Experiments 2 and 3, and we found that call duration was negatively correlated with parasite infection in this population. Thus, we hypothesize that longer calls provide information about male condition, because males that can maintain longer calls have more energy, and thus fewer parasites. However, we also observed females select moderately infected males over uninfected males in Experiment 2, and moderately infected males in Experiment 2 also had longer calls than size-matched uninfected males. While these findings may seem contradictory, the results might instead indicate that males with moderate infections have been successful foragers and therefore have more energy for calling than males with no parasites (potentially unsuccessful foragers), or males with heavy parasite loads. Our results indicate that call duration may be an important factor in female mate choice in this population of Green Treefrogs, and that the relationships between call traits, parasite infection status, and female preference are complex, and ripe for further investigation.

Although our study has uncovered compelling patterns that encourage future study on how oral parasites affect advertisement calls and mate choice in frogs, our results should be viewed within the context of the focal system. Specifically, we were only able to study a single infected population of *D. cinereus*, and acoustic characters and criteria for female mate choice have been shown to vary by population and across geographic distance for Green Treefrogs and other anurans (Asquith et al. 1988; Höbel and Gerhardt 2003). While infection prevalence and parasite abundances per host were very high in our study, they are consistent with prior studies on tongueworm population dynamics in freshwater ponds—for example, Zelmer et al. 1999 recorded mean infection abundances in green frogs (*Lithobates clamitans*) of 2 to 17+ worms per frog, with maximum abundances of 25 to 40+ worms per frog commonly found. Additionally, Wetzel and Esch 1996 tracked tongueworm infections in *L. clamitans* over 3 years and found infection prevalences ranging from 35 to 68% per year, depending on the sex and age class of the host. Therefore, although this study is limited in scope and future work should incorporate multiple populations, we expect that the dynamics uncovered here are likely to be applicable across multiple populations of tongueworms and their frog hosts.

Prospective females must navigate a complex landscape of contrasting signals of male quality, condition, and infection status when choosing a mate. Consistent with sexual selection theory and empirical research (Read 1987; Zuk et al. 1990; Able 1996; Forbes and Robb 2008; Molnár et al. 2013), we find that infection by trophically transmitted internal parasites may be an important factor in mate choice. Although the present study has limitations and uses only natural recorded calls from a single population in the natural environment, it presents uniquely grounded evidence for complex parasite-mediated mate choice decisions in the Green Treefrog *D. cinereus*. Collectively our results suggest that trophically transmitted parasite infections might alter female preferences for established indicators of male quality, like signals of size, condition, or energy levels (e.g., vocalization frequency, call rate) that also correlate with infection abundance, in favor of more subtle traits or combinations of traits that may indicate lower infection severity (e.g., vocalization duration). These unexpected relationships between female choice and male sexual signal traits may show that patterns of mate choice and selection differ in parasitized populations. This study highlights that parasites can interact with host signals in complex ways, requiring further attention in the literature and a nuanced view of parasite-mediated mate choice.

## Supplementary Material

zoag003_Supplementary_Data

## Data Availability

Data and statistical code are available via Figshare (https://doi.org/10.6084/m9.figshare.27321327).

## References

[zoag003-B1] Able DJ , 1996. The contagion indicator hypothesis for parasite-mediated sexual selection. Proc Natl Acad Sci U S A 93(5):2229–2233. 10.1073/pnas.93.5.2229 PMC39940 8700912

[zoag003-B2] Albecker MA, Brantley WB, McCoy MW, 2018. Diet and nematode infection differ between coastal and inland populations of green treefrogs (*Hyla cinerea*). Southeast Nat 17(1):155–165.

[zoag003-B3] Amorim MCP, Vasconcelos RO, Fonseca PJ, 2015. Fish sounds and mate choice. In: Ladich F, editor. Sound Communication of Fishes. Animal Signals and Communication, Vol 4. Vienna: Springer, 1–33.

[zoag003-B4] An D, Waldman B, 2016. Enhanced call effort in Japanese tree frogs infected by amphibian chytrid fungus. Biol Lett 12(3):20160018. 10.1098/rsbl.2016.0018 PMC4843226 26932682

[zoag003-B5] Anderson DR, Burnham KP, White GC, 1998. Comparison of Akaike information criterion and consistent Akaike information criterion for model selection and statistical inference from capture-recapture studies. J Appl Stat 25(2):263–282.

[zoag003-B6] Andersson M , 1994. Sexual Selection. Princeton (NJ): Princeton University Press.

[zoag003-B7] Anikin A , 2019. Soundgen: an open-source tool for synthesizing nonverbal vocalizations. Behav Res Methods 51(2):778–792. 10.3758/s13428-018-1095-7 PMC6478631 30054898

[zoag003-B8] Araya-Salas M, Smith-Vidaurre G, 2017. Warbler: an R package to streamline analysis of animal acoustic signals. Methods Ecol Evol 8(2):184–191.

[zoag003-B9] Asquith A, Altig R, 1990. Male call frequency as a criterion for female choice in *Hyla cinerea*. J Herpetol 24(2):198–201.

[zoag003-B10] Asquith A, Altig R, Zimba P, 1988. Geographic variation in the mating call of the green treefrog *Hyla cinerea*. Am Mid Nat 119(1):101–110.

[zoag003-B11] Augusto-Alves G, Feagles OS, Toledo LF, Höbel G, 2025. Visual cues do not function in a multimodal signalling context for mate attraction in eastern gray treefrogs. Biol J Linn Soc 144(4):blae060.

[zoag003-B12] Balenger SL, Zuk M, 2014. Testing the Hamilton–Zuk hypothesis: past, present, and future. Int Comp Bio 54(4):601–613. 10.1093/icb/icu059 24876194

[zoag003-B13] Bee MA , 2015. Treefrogs as animal models for research on auditory scene analysis and the cocktail party problem. Int J Psychophysiol 95(2):216–237. 10.1016/j.ijpsycho.2014.01.004 PMC4096220 24424243

[zoag003-B14] Bentsen CL, Hunt J, Jennions MD, Brooks R, 2006. Complex multivariate sexual selection on male acoustic signaling in a wild population of *Teleogryllus commodus*. Am Nat 167(4):E102–E116. 10.1086/501376 16670989

[zoag003-B15] Bevier CR, Gorman Gelder AM, 2018. The effect of parasite infection on phonotactic response in the mink frog, *Lithobates septentrionalis*. J Herpetol 52(1):34–39.

[zoag003-B16] Blankers T, Gray DA, Hennig RM, 2017. Multivariate phenotypic evolution: divergent acoustic signals and sexual selection in *Gryllus* field crickets. Evol Biol 44(1):43–55.

[zoag003-B17] Bolek MG, Tracy HR, Janovy Jr J, 2010. The role of damselflies (Odonata: Zygoptera) as paratenic hosts in the transmission of *Halipegus eccentricus* (Digenea: Hemiuridae) to anurans. J Parasitol 96(4):724–735. 10.1645/GE-2365.1 20738199

[zoag003-B18] Bolker B, Bolker MB, R Development Core Team. Package ‘bbmle’. The R Project for Statistical Computing: Vienna, Austria. 2017.

[zoag003-B19] Braicovich PE, Ieno EN, Sáez M, Despos J, Timi JT, 2016. Assessing the role of host traits as drivers of the abundance of long-lived parasites in fish-stock assessment studies. J Fish Biol 89(5):2419–2433. 10.1111/jfb.13127 27592692

[zoag003-B20] Buczek M, Okarma H, Demiaszkiewicz AW, Radwan J, 2016. MHC, parasites and antler development in red deer: no support for the Hamilton & Zuk hypothesis. J Evol Biol 29(3):617–632. 10.1111/jeb.12811 26687843

[zoag003-B21] Buerkle NP, Schrode KM, Bee MA, 2014. Assessing stimulus and subject influences on auditory evoked potentials and their relation to peripheral physiology in green treefrogs (*Hyla cinerea*). Comp Biochem Physiol A Mol Integr Physiol 178:68–81. 10.1016/j.cbpa.2014.08.005 PMC4174320 25151643

[zoag003-B22] Bürkner P-C , 2017. brms: an R package for Bayesian multilevel models using Stan. J Stat Softw. 80(1):1–28.

[zoag003-B23] Byers BE, Kroodsma DE, 2009. Female mate choice and songbird song repertoires. Anim Behav 77(1):13–22.

[zoag003-B24] Cade WH, Wyatt DR, 1984. Factors affecting calling behaviour in field crickets, *Teleogryllus* and *Gryllus* (age, weight, density, and parasites). Behaviour 88(1–2):61–75.

[zoag003-B25] Christensen-Dalsgaard J, Lee N, Bee MA, 2020. Lung-to-ear sound transmission does not improve directional hearing in green treefrogs (*Hyla cinerea*). J Exp Biol 223(20):jeb232421. 10.1242/jeb.232421 32895324

[zoag003-B26] Clayton DH , 1990. Mate choice in experimentally parasitized rock doves: Lousy males lose. Am Zool 30(2):251–262.

[zoag003-B27] Comas M, Ribas A, Milazzo C, Sperone E, Tripepi S, 2014. High levels of prevalence related to age and body condition: host-parasite interactions in a water frog *Pelophylax kl hispanicus*. Acta Herpetol 9(1):25–31.

[zoag003-B28] Connors BM, Hargreaves NB, Jones SRM, Dill LM, 2010. Predation intensifies parasite exposure in a salmonid food chain. J Appl Ecol 47(6):1365–1371.

[zoag003-B29] Creel T, Foster G, Forrester D, 2000. Parasites of the green treefrog, *Hyla cinerea*, from Orange Lake, Alachua County, Florida, U.S.A. Comp Parasitol. 67:255–258.

[zoag003-B30] Delgado RA , 2006. Sexual selection in the loud calls of male primates: signal content and function. Int J Primatol 27(1):5–25.

[zoag003-B31] Drewry GE, Heyer WR, Rand AS, 1982. A functional analysis of the complex call of the frog *Physalaemus pustulosus*. Copeia 1983(3):636–645.

[zoag003-B32] Edler R, Klump GM, Friedl TWP, 2004. Do blood parasites affect reproductive performance in male red bishops (*Euplectes orix*)? A test of the Hamilton-Zuk hypothesis. Ethol Ecol Evol 16(4):315–328.

[zoag003-B33] Edwards DD, Gordon NM, 2021. Helminth diversity of Green Treefrogs (*Hyla cinerea*) in their expanded geographical range. J Parasitol 107(6):923–932. 10.1645/20-6 34902862

[zoag003-B34] Feng AS, Gerhardt HC, Capranica RR, 1976. Sound localization behavior of the green treefrog (*Hyla cinerea*) and the barking treefrog (*H. gratiosa*). J Comp Physiol A 107(3):241–252.

[zoag003-B35] Ferry FRA, Faccini JLH, Inada T, 2011. The role of ear mites of the genus *Raillietia* (Acari: Raillietiidae) in otitis of domestic ruminants. Pesqui Veterinária Bras 31(11):981–984.

[zoag003-B36] Fine ML, Lenhardt ML, 1983. Shallow-water propagation of the toadfish mating call. Comp Biochem Physiol A Physiol 76(2):225–231. 10.1016/0300-9629(83)90319-5 6139203

[zoag003-B37] Fischer J, Kitchen DM, Seyfarth RM, Cheney DL, 2004. Baboon loud calls advertise male quality: acoustic features and their relation to rank, age, and exhaustion. Behav Ecol Sociobiol 56(2):140–148.

[zoag003-B38] Fitch WT, Hauser MD, 1995. Vocal production in nonhuman primates: acoustics, physiology, and functional constraints on “honest” advertisement. Am J Primatol 37(3):191–219. 10.1002/ajp.1350370303 31936952

[zoag003-B39] Forbes MR, Robb T, 2008. Testing hypotheses about parasite-mediated selection using odonate hosts. In: Córdoba-Aguilar A, editor. Dragonflies and Damselflies: Model Organisms for Ecological and Evolutionary Research. New York (NY): Oxford University Press, 175–188.

[zoag003-B40] Garamszegi LZ , 2005. Bird song and parasites. Behav Ecol Sociobiol 59(2):167–180.

[zoag003-B41] Gerhardt HC , 1978. Mating call recognition in the green treefrog (*Hyla cinerea*): the significance of some fine-temporal properties. J Exp Biol 74(1):59–73. 10.1242/jeb.61.1.229 4547332

[zoag003-B42] Gerhardt HC , 1982. Sound pattern recognition in some North American treefrogs (Anura: Hylidae): implications for mate choice. Am Zool 22(3):581–595.

[zoag003-B43] Gerhardt HC , 1987. Evolutionary and neurobiological implications of selective phonotaxis in the green treefrog, *Hyla cinerea*. Anim Behav 35(5):1479–1489.

[zoag003-B44] Gerhardt HC , 1991. Female mate choice in treefrogs: static and dynamic acoustic criteria. Anim Behav 42(4):615–635.

[zoag003-B45] Gerhardt HC , 1994a. The evolution of vocalization in frogs and toads. Annu Rev Ecol Syst 25(1):293–324.

[zoag003-B46] Gerhardt HC , 1994b. Reproductive character displacement of female mate choice in the grey treefrog, *Hyla chrysoscelis*. Anim Behav 47(4):959–969.

[zoag003-B47] Gerhardt HC , 1995. Phonotaxis in female frogs and toads: execution and design of experiments. In: Klump GM, Dooling RJ, Fay RR, Stebbins WC, editors. Methods in Comparative Psychoacoustics. Basel, Switzerland: Springer, 209–220.

[zoag003-B48] Gerhardt HC, Daniel RE, Perrill SA, Schramm S, 1987. Mating behaviour and male mating success in the green treefrog. Anim Behav 35(5):1490–1503.

[zoag003-B49] Gerhardt HC, Huber F, 2002. Acoustic Communication in Insects and Anurans. Chicago (IL): University of Chicago Press.

[zoag003-B50] Gillooly JF, Brown JH, West GB, Savage VM, Charnov EL, 2001. Effects of size and temperature on metabolic rate. Science 293(5538):2248–2251. 10.1126/science.1061967 11567137

[zoag003-B51] Gingras B, Boeckle M, Herbst CT, Fitch WT, 2013. Call acoustics reflect size across four anuran clades. J Zool 289(2):143–150.

[zoag003-B52] Goodnight SR, Blakeslee AMH, McCoy MW, 2025. Genetic diversity and population structure of parasite infrapopulations within and across hosts for two trophically transmitted trematode parasites. PeerJ 13:e19178. 10.7717/peerj.19178 PMC12045269 40313383

[zoag003-B53] Goodnight SR, McCoy MW, 2024. Cannibalism and competition can increase parasite abundance for parasites with complex life history strategies. Ecology 105(7):e4325. 10.1002/ecy.4325 38859696

[zoag003-B54] Greenspan SE, Roznik EA, Schwarzkopf L, Alford RA, Pike DA, 2016. Robust calling performance in frogs infected by a deadly fungal pathogen. Ecol Evol 6(16):5964–5972. 10.1002/ece3.2256 PMC4983606 27547369

[zoag003-B55] Gridi-Papp M, Rand AS, Ryan MJ, 2006. Complex call production in the túngara frog. Nature 441(7089):38–38. 10.1038/441038a 16672962

[zoag003-B56] Hall SR, Sivars-Becker L, Becker C, Duffy MA, Tessier AJ et al, 2007. Eating yourself sick: transmission of disease as a function of foraging ecology. Ecol Lett 10(3):207–218. 10.1111/j.1461-0248.2007.01011.x 17305804

[zoag003-B57] Hamilton WD, Zuk M, 1982. Heritable true fitness and bright birds: a role for parasites? Science 218(4570):384–387. 10.1126/science.7123238 7123238

[zoag003-B58] Hartig F . *DHARMa: Residual diagnostics for hierarchical (multi-level/mixed) regression models.**The R Project for Statistical Computing: Vienna, Austria.* 2020.

[zoag003-B59] Hartig F, Hartig MF. *Package ‘DHARMa’*. The R Project for Statistical Computing: Vienna, Austria. 2017.

[zoag003-B60] Hauser MD , 1993. Rhesus monkey copulation calls: honest signals for female choice? Proc R Soc Lond B Biol Sci 254(1340):93–96. 10.1098/rspb.1993.0132 8290614

[zoag003-B61] Hausfater G, Gerhardt HC, Klump GM, 1990. Parasites and mate choice in Gray Treefrogs, *Hyla versicolor*. Am Zool 30(2):299–312.

[zoag003-B62] Hayward A, Tsuboi M, Owusu C, Kotrschal A, Buechel SD et al, 2017. Evolutionary associations between host traits and parasite load: insights from Lake Tanganyika cichlids. J Evol Biol 30(6):1056–1067. 10.1111/jeb.13053 28187238

[zoag003-B63] Henderson D, Bird DM, Rau ME, Negro JJ, 1995. Mate choice in captive American kestrels, *Falco sparverius*, parasitized by a nematode, *Trichinella pseudospiralis*. Ethology 101(2):112–120.

[zoag003-B64] Höbel G, Gerhardt HC, 2003. Reproductive character displacement in the acoustic communication system of green tree frogs (*Hyla cinerea*). Evolution 57(4):894–904. 10.1111/j.0014-3820.2003.tb00300.x 12778558

[zoag003-B65] Howard RD, Martens RS, Innis SA, Drnevich JM, Hale J, 1998. Mate choice and mate competition influence male body size in Japanese medaka. Anim Behav 55(5):1151–1163. 10.1006/anbe.1997.0682 9632501

[zoag003-B66] Humfeld SC, Grunert B, 2015. Effects of temperature on spectral preferences of female gray treefrogs (*Hyla versicolor*). Herpetol Conserv Biol 10(3):1013–1020.

[zoag003-B67] Hunt J, Breuker CJ, Sadowski JA, Moore AJ, 2009. Male–male competition, female mate choice and their interaction: determining total sexual selection. J Evol Biol 22(1):13–26. 10.1111/j.1420-9101.2008.01633.x 19120810

[zoag003-B68] Jones G , 1997. Acoustic signals and speciation: the roles of natural and sexual selection in the evolution of cryptic species. Adv Study Behav 26:317–354.

[zoag003-B69] Kleindorfer S, Custance G, Peters KJ, Sulloway FJ, 2019. Introduced parasite changes host phenotype, mating signal and hybridization risk: *Philornis downsi* effects on Darwin's finch song. Proc Biol Sci. 286(1904):20190461. 10.1098/rspb.2019.0461 PMC6571467 31185871

[zoag003-B70] Krull WH , 1935. Studies on the life history of *Halipegus occidualis* Stafford, 1905. Am Midl Nat 16(2):129–143.

[zoag003-B71] Labocha MK, Schutz H, Hayes JP, 2014. Which body condition index is best? Oikos 123(1):111–119.

[zoag003-B72] Laird KL, Clements P, Hunter KL, Taylor RC, 2016. Multimodal signaling improves mating success in the green tree frog (*Hyla cinerea*), but may not help small males. Behav Ecol Sociobiol 70(9):1517–1525.

[zoag003-B73] Leary CJ, Crocker-Buta S, Holloway A, Kennedy JGC, 2021. Glucocorticoid-mediated changes in male Green Treefrog vocalizations alter attractiveness to females. Integr Comp Biol 61(1):283–291. 10.1093/icb/icab053 33940612

[zoag003-B74] Lüdecke D , 2018. ggeffects: Tidy data frames of marginal effects from regression models. J Open Source Softw. 3(26):772.

[zoag003-B75] Madelaire CB, José da Silva R, Ribeiro Gomes F, 2013. Calling behavior and parasite intensity in treefrogs, *Hypsiboas prasinus*. J Herpetol 47(3):450–455.

[zoag003-B76] Magnusson A, Skaug H, Nielsen A, Berg C, Kristensen K et al *Package ‘glmmtmb’*. The R Project for Statistical Computing: Vienna, Austria. 2017.

[zoag003-B77] Megía-Palma R, Barrientos R, Gallardo M, Martínez J, Merino S, 2021. Brighter is darker: the Hamilton–Zuk hypothesis revisited in lizards. Biol J Linn Soc 134(2):461–473.

[zoag003-B78] Mikula P, Valcu M, Brumm H, Bulla M, Forstmeier W et al, 2021. A global analysis of song frequency in passerines provides no support for the acoustic adaptation hypothesis but suggests a role for sexual selection. Ecol Lett 24(3):477–486. 10.1111/ele.13662 33314573

[zoag003-B79] Moller AP, Christe P, Lux E, 1999. Parasitism, host immune function, and sexual selection. Q Rev Biol 74(1):3–20. 10.1086/392949 10081812

[zoag003-B80] Molnár O, Bajer K, Mészáros B, Török J, Herczeg G, 2013. Negative correlation between nuptial throat colour and blood parasite load in male European green lizards supports the Hamilton–Zuk hypothesis. Naturwissenschaften 100(6):551–558. 10.1007/s00114-013-1051-4 23644520

[zoag003-B81] Moretti EH, Madelaire CB, Silva RJ, Mendonça MT, Gomes FR, 2014. The relationships between parasite intensity, locomotor performance, and body condition in adult toads (*Rhinella icterica*) from the wild. J of Herp 48(3):277–283.

[zoag003-B82] Moretti EH, Titon B Jr, Madelaire CB, de Arruda R, Alvarez T et al, 2017. Behavioral, physiological and morphological correlates of parasite intensity in the wild Cururu toad (*Rhinella icterica*). Int J Parasitol Parasites Wildl 6(3):146–154. 10.1016/j.ijppaw.2017.06.003 PMC5502792 28725553

[zoag003-B83] Munson AA, Jones C, Schraft H, Sih A, 2020. You’re just my type: mate choice and behavioral types. Trends Ecol Evol 35(9):823–833. 10.1016/j.tree.2020.04.010 32451175

[zoag003-B84] Murphy E, King EA, 2016. Testing the accuracy of smartphones and sound level meter applications for measuring environmental noise. Appl Acoust 106:16–22.

[zoag003-B85] Neelon DP, Rodríguez RL, Höbel G, 2019. On the architecture of mate choice decisions: preference functions and choosiness are distinct traits. Proc R Soc B Biol Sci 286(1897):20182830. 10.1098/rspb.2018.2830 PMC6408907 30963823

[zoag003-B86] Nouri K, Blumstein DT, 2019. Parasites are associated with noisy alarm calls. Front Ecol Evol 7:28.

[zoag003-B87] O’Dwyer K, Dargent F, Forbes MR, Koprivnikar J, 2020. Parasite infection leads to widespread glucocorticoid hormone increases in vertebrate hosts: a meta-analysis. J Anim Ecol 89(2):519–529. 10.1111/1365-2656.13123 31622499

[zoag003-B88] Peig J, Green AJ, 2009. New perspectives for estimating body condition from mass/length data: the scaled mass index as an alternative method. Oikos 118(12):1883–1891.

[zoag003-B89] Perrill SA, Gerhardt HC, Daniel RE, 1982. Mating strategy shifts in male green treefrogs (*Hyla cinerea*): an experimental study. Anim Behav 30(1):43–48.

[zoag003-B90] Pfennig KS, Tinsley RC, 2002. Different mate preferences by parasitized and unparasitized females potentially reduces sexual selection. J Evol Biol 15(3):399–406.

[zoag003-B91] Rand AS, Ryan MJ, 1981. The adaptive significance of a complex vocal repertoire in a neotropical frog. Z Für Tierpsychol 57(3–4):209–214.

[zoag003-B92] R Core Team , 2013. *R: A language and environment for statistical computing*. https://www.r-project.org/

[zoag003-B93] Read AF , 1987. Comparative evidence supports the Hamilton and Zuk hypothesis on parasites and sexual selection. Nature 328(6125):68–70.

[zoag003-B94] Reichert MS, Bolek MG, McCullagh EA, 2023. Parasite effects on receivers in animal communication: hidden impacts on behavior, ecology, and evolution. Proc Natl Acad Sci U S A 120(30):e2300186120. 10.1073/pnas.2300186120 PMC10372545 37459523

[zoag003-B95] Robillard T, Höbel G, Carl Gerhardt H, 2006. Evolution of advertisement signals in North American hylid frogs: vocalizations as end-products of calling behavior. Cladistics 22(6):533–545. 10.1111/j.1096-0031.2006.00118.x 34892895

[zoag003-B96] Ryan MJ , 1980. Female mate choice in a neotropical frog. Science 209(4455):523–525. 10.1126/science.209.4455.523 17831371

[zoag003-B97] Ryan MJ , 1983. Sexual selection and communication in a neotropical frog, *Physalaemus pustulosus*. Evolution 37(2):261–272. 10.1111/j.1558-5646.1983.tb05536.x 28568357

[zoag003-B98] Ryan MJ , 1990. Signals, species, and sexual selection. Am Sci 78(1):46–52.

[zoag003-B99] Ryan MJ, Guerra MA, 2014. The mechanism of sound production in túngara frogs and its role in sexual selection and speciation. Curr Opin Neurobiol 28:54–59. 10.1016/j.conb.2014.06.008 25033110

[zoag003-B100] Ryan MJ, Keddy-Hector A, 1992. Directional patterns of female mate choice and the role of sensory biases. Am Nat 139:S4–S35.

[zoag003-B101] Ryan MJ, Rand AS, 2003. Sexual selection in female perceptual space: how female tungara frogs perceive and respond to complex population variation in acoustic mating signals. Evolution 57(11):2608–2618. 10.1111/j.0014-3820.2003.tb01503.x 14686535

[zoag003-B102] Sanchez CA, Becker DJ, Teitelbaum CS, Barriga P, Brown LM et al, 2018. On the relationship between body condition and parasite infection in wildlife: a review and meta-analysis. Ecol Lett 21(12):1869–1884. 10.1111/ele.13160 30369000

[zoag003-B103] Schneider CA, Rasband WS, Eliceiri KW, 2012. NIH Image to ImageJ: 25 years of image analysis. Nat Methods 9(7):671–675. 10.1038/nmeth.2089 PMC5554542 22930834

[zoag003-B104] Searcy WA, Andersson M, 1986. Sexual selection and the evolution of song. Annu Rev Ecol Syst 17(1):507–533.

[zoag003-B105] Shangi NE, Gardner KM, Mennill DJ, Doucet SM, 2022. Is color related to parasite load in a sexually dichromatic neotropical toad? Herp 78(4):235–243.

[zoag003-B106] Stigge HA, Bolek MG, 2016. Anuran host species influences site fidelity of *Halipegus occidualis*. J Parasitol 102(1):47–53. 10.1645/15-790 26412569

[zoag003-B107] Thomas LJ , 1939. Life cycle of a fluke, *Halipegus eccentricus* n. sp., found in the ears of frogs. J Parasitol 25(3):207–221.

[zoag003-B108] Van Wert JC, Mensinger AF, 2019. Seasonal and daily patterns of the mating calls of the oyster toadfish, *Opsanus tau*. Biol Bull 236(2):97–107. 10.1086/701754 30933642

[zoag003-B109] Vehtari A, Gelman A, Gabry J, 2017. Practical Bayesian model evaluation using leave-one-out cross-validation and WAIC. Stat Comput 27(5):1413–1432.

[zoag003-B110] Weatherhead PJ , 1990. Secondary sexual traits, parasites, and polygyny in red-winged blackbirds, *Agelaius phoeniceus*. Behav Ecol 1(2):125–130.

[zoag003-B111] Wetzel EJ, Esch GW, 1996. Seasonal population dynamics of *Halipegus occidualis* and *Halipegus eccentricus* (Digenea: Hemiuridae) in their amphibian host, *Rana clamitans*. J Parasitol 82(3):414–422. 8636845

[zoag003-B112] Wickham H , 2011. ggplot2. Wiley Interdiscip Rev Comput Stat. 3(2):180–185.

[zoag003-B113] Wilczynski W, Zakon HH, Brenowitz EA, 1984. Acoustic communication in spring peepers. J Comp Physiol A 155(5):577–584.

[zoag003-B114] Worden BD, Parker PG, Pappas PW, 2000. Parasites reduce attractiveness and reproductive success in male grain beetles. Anim Behav 59(3):543–550. 10.1006/anbe.1999.1368 10715176

[zoag003-B115] Zelmer DA, Esch GW, 1998. Bridging the gap: the odonate naiad as a paratenic host for *Halipegus occidualis* (Trematoda: Hemiuridae). J Parasitol 84(1):94–96. 9488344

[zoag003-B116] Zelmer DA, Esch GW, 2000. Relationship between structure and stability of a *Halipegus occidualis* component population in green frogs: a test of selective treatment. J Parasitol 86(2):233–240. 10.1645/0022-3395(2000)086[0233:RBSASO]2.0.CO;2 10780538

[zoag003-B117] Zelmer DA, Wetzel EJ, Esch GW, 1999. The role of habitat in structuring *Halipegus occidualis* metapopulations in the green frog. J Parasitol 85(1):19–24. 10207357

[zoag003-B118] Zhu B, Zhou Y, Yang Y, Deng K, Wang T et al, 2021. Multisensory modalities increase working memory for mating signals in a treefrog. J Anim Ecol 90(6):1455–1465. 10.1111/1365-2656.13465 33666233

[zoag003-B119] Ziegler L, Arim M, Bozinovic F, 2016. Intraspecific scaling in frog calls: the interplay of temperature, body size and metabolic condition. Oecologia 181(3):673–681. 10.1007/s00442-015-3499-8 26552381

[zoag003-B120] Zuk M , 1992. The role of parasites in sexual selection: current evidence and future directions. Adv Study Behav 21:39–68.

[zoag003-B121] Zuk M, Thornhill R, Ligon JD, Johnson K, 1990. Parasites and mate choice in red jungle fowl. Am Zool 30(2):235–244. 10.1111/j.1558-5646.1990.tb05933.x 28567977

