# Supplementary material for: Oral parasites alter male advertisement signals and female mate choice in the Green Treefrog (Dryophytes cinereus)
Source: Curr Zool. 2026 Jan 22;72(5):731–46. doi: 10.1093/cz/zoag003 (PMC13690587; doi:10.1093/cz/zoag003)
Supplement: Supplementary file 1 [file zoag003_supplementary_data.zip › Appendix_SupplementaryFigures_CurrentZoology_revised.docx]

**Supplemental Figure S1. Relationships between male Green Treefrog body mass (in g) and call characteristics.** Call duration in sec (panel A), high and low dominant frequencies in kHz (panels B and C), pulse structure (panels D, E, and F), and call rate in calls/sec (panel G) are shown. Points are raw data and plotted lines are ggplot2 linear models.

**Supplemental Figure S2. Relationships between male Green Treefrog snout vent length (SVL, in mm) and call characteristics.** Call duration in sec (panel A), high and low dominant frequencies in kHz (panels B and C), pulse structure (panels D, E, and F), and call rate in calls/sec (panel G) are shown. Points are raw data and plotted lines are ggplot2 linear models.

**Supplementary Figure S3. Effects of temperature on call rate and pulse duration for all frogs (n=177) and infected frogs only (n=72)**. Predicted values (colored lines) are plotted over raw data on the left of each panel, and error envelopes are 95% credible intervals. Coefficient estimates from each model for parasite abundance are on the log scale in the rightmost column with 95% credible interval error bars.

**Supplementary Figure S4.** Relationships between parasite infection load and mate choice for playback Experiment 1 (A) and Experiment 2 (B). Plotted lines are predicted relationships from binomial generalized linear mixed models and error envelopes are 95% confidence intervals, plotted over raw data. The y-axis is the proportion of trials each male call was selected over the competitor for each experiment.
