# Supplementary material for: Oral parasites alter male advertisement signals and female mate choice in the Green Treefrog (Dryophytes cinereus)
Source: Curr Zool. 2026 Jan 22;72(5):731–46. doi: 10.1093/cz/zoag003 (PMC13690587; doi:10.1093/cz/zoag003)
Supplement: Supplementary file 1 [file zoag003_supplementary_data.zip › Appendix_SupplementaryTables_CurrentZoology.docx]

**Supplementary Table S1. GLMMs and corresponding weights for tongueworm infection abundance analysis.** Sample size corrected Akaike Information Criterion (AICc) model comparisons were used to determine best fit. Distribution codes are LNB-ZI = zero-inflated linear negative binomial, QNB-ZI = zero-inflated quadratic negative binomial, and P-ZI = zero-inflated Poisson.

| **Fixed effects** | **Distribution** | **dLogLik** | **dAIC** | **df** | **weight** |
| --- | --- | --- | --- | --- | --- |
| log(SVL) + Month | LNB-ZI | 61 | 0.0 | 10 | 0.33 |
| log(SVL) + Month | QNB-ZI | 60.9 | 0.3 | 10 | 0.28 |
| log(SVL) | LNB-ZI | 57.7 | 0.7 | 7 | 0.23 |
| log(SVL) | QNB-ZI | 57.3 | 1.4 | 7 | 0.16 |
| Month | LNB-ZI | 53.5 | 13 | 9 | <0.001 |
| Month | QNB-ZI | 53.2 | 13.7 | 9 | <0.001 |
| NULL | QNB-ZI | 49.8 | 14.6 | 6 | <0.001 |
| NULL | LNB-ZI | 49.8 | 14.6 | 6 | <0.001 |
| log(SVL) + Month | P-ZI | 24.2 | 71.7 | 9 | <0.001 |
| log(SVL) | P-ZI | 14.4 | 85.3 | 6 | <0.001 |
| Month | P-ZI | 11.1 | 95.8 | 8 | <0.001 |
| NULL | P-ZI | 0.0 | 112.1 | 5 | <0.001 |

**Supplementary Table S2. Model coefficient estimates and credible intervals of Bayesian mixed-effects models for each call characteristic from analyses including all males (n=177).** Estimates and credible intervals presented on the log scale. Distribution codes are SkN = skew-normal, N = normal.

| **­­Response** | **Model** | **Intercept** | **Parasite estimate** | **Temperature estimate** | **Distribution** |
| --- | --- | --- | --- | --- | --- |
| **High dominant frequency (kHz)** | offset(log(SVL)) +  log(mature worms) + (1\|frogID) | **-2.70,**  (-2.72 – -2.68) | **-0.017,**  (-0.04 – 0.01) | **__** | SkN |
| **Low dominant frequency (kHz)** | offset(log(SVL)) + log(mature worms) + (1\|frogID) | **-3.856,**  (-3.88 – -3.84) | **-0.015,**  (-0.04 – 0.01) | **__** | SkN |
| **Call duration**  **(ms)** | offset(log(SVL)) + log(mature worms) + (1\|frogID) | **-2.211,**  (-2.22 – -2.19) | **-0.003,**  (-0.002 – 0.000) | **__** | SkN |
| **Pulse duration**  **(ms)** | offset(log(SVL)) + temperature + log(mature worms) + (1\|frogID) | **-2.548,**  (-2.17 – -2.38) | **-0.008,**  (-0.02 – 0.01) | **-0.00004,**  (-0.02 – 0.01) | SkN |
| **Pulse number**  **(per call)** | offset(log(SVL)) +  log(mature worms) + (1\|frogID) | **-0.237,**  (-0.28 – -0.19) | **-0.021,**  (-0.06 – 0.02) | **__** | SkN |
| **Pulse rate**  **(pulses/ms)** | offset(log(SVL)) +  log(mature worms) + (1\|frogID) | **-5.105,**  (-5.17 – -5.04) | **-0.068,**  (-0.11 – -0.03) | **__** | SkN |
| **Call rate**  **(calls/sec)** | offset(log(SVL)) + temperature + log(mature worms) + (1\|frogID) | **-4.373,**  (-4.60 – -4.15) | **-0.025,**  (-0.05 – -0.005) | **0.040,**  (0.03 – 0.05) | N |

**Supplementary Table S3. Model coefficient estimates and credible intervals of Bayesian mixed-effects models for each call characteristic from analyses including only infected males (n=72).** Estimates and credible intervals presented on the log scale. Distribution codes are SkN = skew-normal.

| **­­Response** | **Model** | **Intercept** | **Parasite estimate** | **Temperature estimate** | **Distribution** |
| --- | --- | --- | --- | --- | --- |
| **High dominant frequency (kHz)** | offset(log(SVL)) +  log(mature worms) + (1\|frogID) | **-2.65,**  (-2.70 – -2.60) | **-0.048,**  (-0.08 – -0.01) | **__** | SkN |
| **Low dominant frequency (kHz)** | offset(log(SVL)) + log(mature worms) + (1\|frogID) | **-3.817,**  (-3.87 – -3.77) | **-0.041,**  (-0.07 – -0.004) | **__** | SkN |
| **Call duration**  **(ms)** | offset(log(SVL)) + log(mature worms) + (1\|frogID) | **-2.193,**  (-2.22 – -2.16) | **-0.004,**  (-0.01 – 0.000) | **__** | SkN |
| **Pulse duration**  **(ms)** | offset(log(SVL)) + temperature + log(mature worms) + (1\|frogID) | **2.380,**  (-2.64 – -2.14) | **0.003,**  (-0.02 – 0.24) | **-0.010,**  (-0.02 – 0.003) | SkN |
| **Pulse number**  **(per call)** | offset(log(SVL)) +  log(mature worms) + (1\|frogID) | **-0.145,**  (-0.22 – -0.06) | **-0.080,**  (-0.14 – -0.02) | **__** | SkN |
| **Pulse rate**  **(pulses/ms)** | offset(log(SVL)) +  log(mature worms) + (1\|frogID) | **-5.105,**  (-5.17 – -5.04) | **-0.068,**  (-0.11 – -0.03) | **__** | SkN |
| **Call rate**  **(calls/sec)** | offset(log(SVL)) + temperature + log(mature worms) + (1\|frogID) | **-4.494,**  (-4.87 – -4.11) | **-0.0278,**  (-0.06 – 0.002) | **0.044,**  (0.03 – 0.06) | N |

**Top models (GLMMs) and corresponding weights for our second approach to the female choice analysis for the three playback experiments.** In all cases, the response variable was the number of trials a male was selected by a female, with the number of times he was not selected as the binomial denominator [of the form (number selections, number rejections)]. Sample size corrected Akaike Information Criterion (AICc) model comparisons were used to determine best fit. All models were fit with binomial error distributions and logit link functions.

# Supplementary Table S4. Top models for Experiment 1.

| Fixed Effects | dLogLik | dAIC | df | weight |
| --- | --- | --- | --- | --- |
| highfreq + worms | 19.1 | 0.0 | 4 | 0.1890 |
| worms + call duration * highfreq | 20.8 | 0.5 | 6 | 0.1439 |
| highfreq * call duration | 19.5 | 1.0 | 5 | 0.1121 |
| highfreq + call duration + worms | 19.5 | 1.2 | 5 | 0.1035 |
| highfreq * worms | 19.3 | 1.5 | 5 | 0.0892 |
| highfreq + worms + call rate | 19.1 | 1.9 | 5 | 0.0719 |
| highfreq + call duration | 18.0 | 2.1 | 4 | 0.0657 |
| worms + call duration + highfreq + lowfreq | 19.6 | 3.0 | 6 | 0.0426 |
| worms * highfreq + call duration | 19.5 | 3.1 | 6 | 0.0407 |
| worms * lowfreq | 18.5 | 3.1 | 5 | 0.0406 |
| highfreq + call duration + worms + call rate | 19.5 | 3.1 | 6 | 0.0404 |
| highfreq | 16.2 | 3.7 | 3 | 0.0296 |
| worms | 15.5 | 5.2 | 3 | 0.0139 |
| worms + call duration | 15.6 | 6.8 | 4 | 0.0062 |
| worms * call duration | 16.5 | 7.0 | 5 | 0.0056 |
| worms + call rate | 15.5 | 7.1 | 4 | 0.0053 |
| call duration | 1.3 | 33.6 | 3 | <0.001 |
| null | 0.0 | 34.1 | 2 | <0.001 |
| call rate + call duration | 1.3 | 35.6 | 4 | <0.001 |
| lowfreq | 0.1 | 36.0 | 3 | <0.001 |
| call rate | 0.0 | 36.1 | 3 | <0.001 |

**Supplementary Table S5.** Top models for Experiment 2.

| Fixed Effects | dLogLik | dAIC | df | weight |
| --- | --- | --- | --- | --- |
| worms + call duration * highfreq | 20.7 | 0.0 | 6 | 0.3017 |
| worms + call duration + highfreq | 19.1 | 1.2 | 5 | 0.1659 |
| worms * highfreq + call duration | 20.1 | 1.2 | 6 | 0.1617 |
| worms + call duration + highfreq + call rate | 19.6 | 2.4 | 6 | 0.0914 |
| highfreq + worms + call rate | 18.5 | 2.5 | 5 | 0.0859 |
| worms * highfreq | 18.3 | 3.0 | 5 | 0.0684 |
| worms + call duration | 16.7 | 4.1 | 4 | 0.0382 |
| worms | 15.6 | 4.4 | 3 | 0.0338 |
| worms * call duration | 17.2 | 5.2 | 5 | 0.0227 |
| worms + call rate | 15.6 | 6.4 | 4 | 0.0125 |
| highfreq * call duration | 16.3 | 6.8 | 5 | 0.0099 |
| worms * lowfreq | 16.0 | 7.5 | 5 | 0.0069 |
| highfreq + call duration | 12.8 | 11.9 | 4 | <0.001 |
| call duration | 9.8 | 15.9 | 3 | <0.001 |
| call rate + call duration | 9.9 | 17.7 | 4 | <0.001 |
| highfreq | 8.4 | 18.7 | 3 | <0.001 |
| null | 5.5 | 22.4 | 2 | <0.001 |
| call rate | 5.6 | 24.3 | 3 | <0.001 |
| lowfreq | 0.0 | 35.5 | 3 | <0.001 |
| worms + highfreq | 0.0 | 37.5 | 4 | <0.001 |
| worms + call duration + highfreq + lowfreq | 0.0 | 41.5 | 6 | <0.001 |

**Supplementary Table S6.** Top models for Experiment 3.

| Fixed Effects | dLogLik | dAIC | df | weight |
| --- | --- | --- | --- | --- |
| call rate + call duration | 14.8 | 0.0 | 4 | 0.7388 |
| call rate | 12.1 | 3.4 | 3 | 0.1375 |
| worms + call rate | 12.1 | 5.4 | 4 | 0.0505 |
| highfreq + worms + call rate | 12.2 | 7.1 | 5 | 0.0209 |
| call duration | 9.5 | 8.6 | 3 | 0.0101 |
| worms * highfreq + call duration | 12.0 | 9.4 | 6 | 0.0067 |
| highfreq + call duration | 10.0 | 9.6 | 4 | 0.0062 |
| worms+call duration + highfreq | 10.6 | 10.2 | 5 | 0.0044 |
| highfreq * call duration | 10.6 | 10.3 | 5 | 0.0043 |
| worms + call duration | 9.5 | 10.5 | 4 | 0.0039 |
| worms * lowfreq | 10.5 | 10.5 | 5 | 0.0039 |
| highfreq + worms | 9.2 | 11.2 | 4 | 0.0027 |
| worms + call duration + highfreq + lowfreq | 11.1 | 11.3 | 6 | 0.0026 |
| worms * highfreq | 10.0 | 11.4 | 5 | 0.0024 |
| worms * call duration | 10.0 | 11.4 | 5 | 0.0024 |
| lowfreq | 7.1 | 13.3 | 3 | <0.001 |
| highfreq | 6.9 | 13.6 | 3 | <0.001 |
| null | 5.7 | 14.2 | 2 | <0.001 |
| worms | 5.7 | 16.1 | 3 | <0.001 |
| highfreq + call duration + worms + call rate | 0.0 | 33.5 | 6 | <0.001 |
